# Eine Kontroverse auf Tape: Oral History, Zeug:innenschaft und Mythenbildung in der US-amerikanischen Gentechnikdebatte (1975–1980)

**DOI:** 10.1007/s00048-025-00411-x

**Published:** 2025-03-25

**Authors:** Christoffer Leber

**Affiliations:** DFG-Forschungsgruppe: Kooperation und Konkurrenz in den Wissenschaften, Lehrstuhl für Wissenschaftsgeschichte, Historisches Seminar, Ludwig-Maximilians-Universität München, München, Deutschland

**Keywords:** Gentechnik, Rekombinante DNA, Asilomar-Konferenz, Cambridge-Kontroverse, Oral History, Mythenbildung, Genetic engineering, Recombinant DNA, Asilomar conference, Cambridge controversy, Oral history, Mythmaking

## Abstract

In den 1970er Jahren gewann die Oral History in der Wissenschaftsgeschichte zunehmend an Bedeutung. Ein Beispiel für diesen Trend ist die Recombinant DNA History Collection, die der Wissenschaftshistoriker Charles Weiner (1932–2012) 1975 am MIT aufbaute. Die Sammlung folgte dem Anspruch einer „history in the making“, also wissenschaftshistorisch bedeutende Ereignisse im Werden zu dokumentieren. Sie umfasste Zeitzeug:inneninterviews, Ton- und Videoaufnahmen, Presseausschnitte und weiteres Archivmaterial zum Thema rekombinante DNA (rDNA). Neben der Asilomar-Konferenz 1975 stand die Kontroverse um rDNA in Cambridge, Massachusetts 1976/77 im Fokus der Sammlung. Als im Frühjahr 1976 bekannt wurde, dass Harvard den Bau eines P3-Labors für rDNA-Experimente plante, berief der Stadtrat von Cambridge zwei öffentliche Anhörungen ein, bildete ein Bürgerkomitee und verabschiedete die erste lokale Verordnung zur Regulierung von rDNA-Forschung in den USA. Im Folgenden argumentiere ich, dass die Oral History Collection am MIT einerseits das aufklärerische Ziel verfolgte, den Mythos einer neutralen, objektiven und wertfreien Wissenschaft zu widerlegen, andererseits jedoch selbst zur Schaffung neuer Mythen beitrug.

## Abstract (en)

In the 1970s, historians discovered oral history as a new approach to the history of modern science. One example of this trend is the Recombinant DNA History Collection, which was created by Charles Weiner (1932–2012) at MIT in 1975. Following the approach of “history in the making”, the collection aimed to document events that were considered turning points in the history of science. The collection included oral history interviews, audio and video recordings, press clippings, and further material around recombinant DNA (rDNA). In addition to the 1975 Asilomar Conference, the collection focused on the controversy about rDNA research in Cambridge (MA) in 1976/77. When Mayor Alfred Vellucci heard from Harvard’s plan to build a laboratory for rDNA experiments on a P3 containment level, he convened two public hearings at the City Council in the summer of 1976. After a critical assessment of potential hazards of rDNA research by a citizen board, Cambridge passed the first local ordinance for the regulation rDNA research in the United States. In the following, I argue that the Recombinant DNA History Collection challenged the myth of neutral, objective, and disinterested science. Yet at the same time, it created new myths.

## Einleitung

Im November 2019, wenige Monate vor dem Ausbruch der Covid-19-Pandemie, feierte die Dokumentation *From Controversy to Cure: Inside the Cambridge Biotech Boom* ihre Premiere am Massachusetts Institute of Technology (MIT).^1^ Produziert und gedreht wurde die Dokumentation von dem Wissenschaftsjournalisten Joe McMaster, der sich bereits in mehreren Filmen mit der Geschichte des MIT auseinandergesetzt hatte.^2^*From Controversy to Cure* zeigt den Wandel des Kendall Square in Cambridge (USA) von einer unbebauten, menschenleeren Industriebrache am Fuße des Charles River hin zum dichtesten Biotech-Zentrum der Welt (McMaster 2019; Buderi 2022). Dass der Kendall Square zum globalen Zentrum der Biotech-Industrie wurde, war das unerwartete Ergebnis rechtlicher, forschungspolitischer und stadtplanerischer Faktoren. Ursprünglich hatte die NASA nämlich vor, das Gelände für eigene Forschungszwecke im *Space Race* gegen die Sowjetunion zu nutzen (vgl. *MIT News* 2019). In ihrem Aufbau gleicht die Dokumentation einer antiken Tragödie: Sie beginnt mit dem rasanten Aufstieg der Molekularbiologie in der Nachkriegszeit, Nixons „War on Cancer“-Kampagne und dem Bau des Center for Cancer Research am MIT unter der Leitung Salvador Lurias (1912–1991), der aus dem faschistischen Italien in die USA emigriert war (Selya 2022). Das Center, so heißt es, avancierte schnell zu einem *Powerhouse* der internationalen Krebsforschung und brachte vier Nobelpreisträger hervor: David Baltimore, Salvador Luria, Susumu Tonegawa und Phillip A. Sharp. Dank neuer molekularbiologischer Verfahren folgte eine Entdeckung auf die nächste und man war sich sicher, die genetischen und viralen Ursachen von Krebs bald zu entschlüsseln. Die Frage, ob Krebs durch Viren verursacht wird, ob er ansteckend sei und ob man sich gegen ihn impfen lassen könne, war in der biomedizinischen Forschung der 1970er *das* brennende Thema (Scheffler 2019). Eine Legende von damals besagt, dass auf dem Stockwerk des Cancer Center immer das Licht gebrannt habe (vgl. McMaster 2019).

Doch auf den Höhenflug folgte die Krise: Die Entwicklung der rekombinanten DNA (rDNA) – ein molekularbiologisches In-Vitro-Verfahren, bei dem DNA-Moleküle isoliert und in einen Modellorganismus eingeschleust wurden – rief eine öffentliche Kontroverse um mögliche Gefahren für Mensch und Umwelt hervor. Eine Kontroverse, welche die rekombinante Forschung am MIT und in Harvard beinahe zum Erliegen gebracht hätte. Welche potenziellen Risiken und Gefahren barg die neue Technologie? Wie sollte rDNA-Forschung reguliert werden, und wer sollte sie regulieren? War es ethisch, Experimente dieser Art durchzuführen? Und wie wahrscheinlich war es, dass sie für kommerzielle oder gar militärische Zwecke missbraucht wurden? In dem Kapitel „Frankenbugs?“ zeichnet die Dokumentation den Verlauf der Kontroverse in Wissenschaft, Politik und Öffentlichkeit nach. Obwohl sich Biolog:innen zunächst auf ein internationales Moratorium verständigt und auf der Asilomar-Konferenz 1975 Maßnahmen zur Regulierung von rDNA-Experimenten erarbeitet hatten, kam es in verschiedenen Universitätsstädten zu lokalen Protesten gegen rekombinante Forschung. Im Sommer 1976 erreichte die Kontroverse auch Cambridge, wo der umstrittene Bau eines P3-Labors für rDNA-Experimente an der Harvard University hitzige Auseinandersetzungen zwischen Akteur:innen aus Wissenschaft, Politik und lokaler Öffentlichkeit provozierte (Botelho 2019; Durant 2012; Feldman & Lowe 2008).^3^ Nach zwei Anhörungen vor dem Stadtrat von Cambridge und einem Bürgerkomitee, das potenzielle Risiken evaluierte, verabschiedete Cambridge 1977 die erste lokale Verordnung zur Regulierung von rDNA-Experimenten in den USA. Diese Verordnung bereitete den Boden für den Biotech-Boom in Cambridge rund um den Kendall Square. Aus der Krise wurde ein Welterfolg, so die Botschaft der Dokumentation: „Join us for the story of how the unlikely mix of science and engineering, politics, the space race, and urban renewal transformed Kendall Square into the ‚biotechnology capital of the world‘“, heißt es in der Ankündigung.^4^

Wie die Dokumentation suggeriert, gehört die Cambridge-Kontroverse heute zu den dramatischsten Kapiteln in der Geschichte der Gentechnik (vgl. Botelho 2019; Cobb 2022; Durant 2012; Feldman & Lowe 2008; Krimsky 1982; Wright 1994). Dass die Anhörungen vor dem Stadtrat in Cambridge so gut dokumentiert sind, verdankt sich nicht nur der ausführlichen Presseberichterstattung, sondern auch einer Oral-History-Sammlung, die der Wissenschaftshistoriker Charles Weiner 1975 am MIT aufbaute: Die Recombinant DNA History Collection. Neben Thomas Kuhns (1922–1996) Projekt Sources for History of Quantum Physics war die MIT-Sammlung eine der ersten und größten ihrer Art in den Vereinigten Staaten (vgl. Doel 2003: 356). Weiners Sammlung verstand sich als dokumentarisches und aufklärerisches Projekt zugleich: Einerseits hatte sie zum Ziel, Wissenschaftsgeschichte in *statu nascendi* zu dokumentieren, andererseits sollte sie den Mythos einer objektiven, neutralen und wertfreien Wissenschaft widerlegen (Dorman 1980: 86ff.; Radkau 1988: 337). Die Sammlung wurde ein voller Erfolg und lieferte die Quellenbasis für zahlreiche Studien zur Gentechnikdebatte in den USA und Europa (Cobb 2022; Gottweis 1998; Krimsky 1982; Wright 1994).

Wie ich im Folgenden argumentiere, hatte die Sammlung trotz ihrer aufklärerischen Funktion einen Nebeneffekt: Sie trug zur Schaffung neuer Mythen bei, indem sie die Cambridge-Kontroverse ins Zentrum der amerikanischen rDNA-Debatte rückte und damit zu etwas Größerem machte, als sie eigentlich war. Während die Episode in zahlreichen Abhandlungen als dramatischer Höhepunkt der rDNA-Debatte und *role model* für öffentliche Partizipation überhöht wurde, waren die Ereignisse in Wirklichkeit weniger revolutionär. Neben Charles Weiner waren es Ruth Hubbard, Sheldon Krimsky, Dorothy Nelkin und Susan Wright, die in ihren wegweisenden Arbeiten zur rDNA-Debatte bestehende Biases reproduzierten und zur Mythenbildung um Cambridge beitrugen. Hinzu kamen die zeitgenössische Presse, Organisationen wie Science for the People und schillernde Politiker wie Alfred Vellucci (1915–2002), die ihren eigenen Beitrag zur Mythologisierung der Cambridge-Episode leisteten.

Analytisch nähere ich mich der Cambridge-Kontroverse über den Begriff des „politischen Mythos“ an. In Anlehnung an Frank Becker verstehe ich unter „politischem Mythos“ eine selektive, idealisierte Narration der Vergangenheit, die eine sinn- und identitätsstiftende Funktion für die Gegenwart hat (Becker 2005). Welche Bedeutung Gründungsmythen für die Legitimation wissenschaftlicher Disziplinen haben, untersuchte Pnina Abir-Am am Beispiel der Molekularbiologie. Neben den Erinnerungen der „Gründungsväter“ der Molekularbiologie fungierte die Historiografie als Legitimation zweiter Ordnung, indem sie die Wirkungsorte, Autoritäten, Entdeckungen und Durchbrüche in eine teleologische, notwenige Erfolgsgeschichte einbettete (Abir-Am 1985: 74f.; 105ff.). Besonders in Autobiografien versuchten Molekularbiologen die Kontrolle über ihr eigenes Narrativ und das ihrer Disziplin zu behalten; einige kleideten ihr Leben in die Geschichte eines Helden, der in der Nachkriegszeit vom unangepassten „Underdog“ zum „Wissenschaftsstar“ aufstieg (Abir-Am 1991: 327). Für die Co-Produktion von Geschichtsschreibung, Mythen und Erinnerungsorten ist die Molekularbiologie ein instruktives Beispiel.

Mein Aufsatz gliedert sich in fünf Kapitel: Er beginnt mit einem kurzen Überblick über die Geschichte der Oral History und deren methodische Relevanz für die Wissenschaftsgeschichte. Im Anschluss rekonstruiere ich die Gentechnikdebatte in den USA und die Kontroverse um rDNA-Forschung in der Boston Area. Das darauffolgende Kapitel widmet sich der Entstehungsgeschichte der Recombinant DNA History Collection und ihrer Bedeutung für die in den 1970er Jahren eingerichteten Technology Studies am MIT. Im vierten Kapitel untersuche ich die Oral-History-Interviews, die Weiner und sein Team mit Zeitzeug:innen der rDNA-Debatte führten, sowie den Lehrfilm *Hypothetical Risk* über die öffentliche Anhörung vor dem Stadtrat von Cambridge im Sommer 1976. Im Zentrum des letzten Kapitels steht die Frage: Inwiefern trug die Oral-History-Sammlung zur Schaffung neuer Mythen bei?

## Oral History und Wissenschaftsgeschichte

Die Oral History – ein Instrumentarium zur mündlichen Erfragung und Aufzeichnung von historischen Erfahrungen, Wahrnehmungen und Deutungen – ist heute kaum mehr aus der Geschichtswissenschaft wegzudenken (vgl. Abrahams 2016; Althaus & Apel 2023; Ritchie 2012, 2014; Thompson 1978). Obwohl die mündliche Überlieferung von Geschichte bereits in der Antike gängige Praxis war, wurde die Oral History in unserem modernen, westlichen Verständnis in der zweiten Hälfte des 20. Jahrhunderts geprägt (Althaus & Apel 2023: 6). Für die Entwicklung der Oral History in der Nachkriegszeit waren zwei Entwicklungen ausschlaggebend: zum einen das Interesse der Sozialgeschichte an einer Geschichte „von unten“ und deren Hinwendung zur Lebenswelt marginalisierter Gruppen, zum anderen die Entwicklung neuer Kommunikationstechnologien wie Tonbandgerät oder Filmkamera (de Chadarevian 1997: 54f.).

Die Oral History wurde besonders in den USA und Großbritannien methodisch weiterentwickelt. Der Journalist und Historiker Allan Nevins (1890–1971), seit 1939 Professor für Geschichte an der Columbia University, gründete 1948 das Columbia Center for Oral History Research und schuf damit das erste Archiv zur Sammlung und Speicherung von Oral-History-Interviews.^5^ In seinem Hauptwerk *The Gateway to History* (1938) diskutierte Nevins verschiedene Ansätze, Methoden und Quellen der Geschichtsschreibung seit Thukydides. Nevins verfolgte das Ziel einer „People’s History“, die nicht nur auf physischen Überresten, Manuskripten, Inschriften, Büchern und Ideen aufbaute, sondern auch auf den Stimmen einflussreicher Persönlichkeiten aus Politik, Kultur und Wirtschaft (Nevins 1938: 117; te Heesen 2022: 67f.). Nur so könne man ihm zufolge die Stimmung, den Geist einer vergangenen Epoche einfangen und den leblosen Dokumenten Lebendigkeit einhauchen (Nevins 1938: 117). In ihrer Ausrichtung blieb die Oral-History-Sammlung an der Columbia University dem Eliteninterview verpflichtet, denn als Interviewpartner wurden die „movers and shakers of society“ ausgewählt (Doel 2003: 356; te Heesen 2021, 2022).

Analog zur Sozialgeschichte „von unten“ setzte sich auch in der Oral History eine Richtung durch, die marginalisierten Gruppen (wie z. B. versklavten Menschen) oder der Arbeiterschicht eine Stimme zu geben versuchte. Damit ist eine politische Stoßrichtung angesprochen, die seit den 1970er Jahren mit der Oral History verbunden ist: die Demokratisierung von Geschichtsschreibung und -deutung (Althaus & Apel 2023). Indem die Oral History marginalisierten Gruppen Sichtbarkeit verlieh, fungierte sie als Korrektiv für hegemoniale Geschichtsdeutungen. Lutz Niethammer, ein führender Kenner der Oral History in Deutschland, schrieb dazu: „Eine demokratische Zukunft bedarf einer Vergangenheit, in der nicht nur die Oberen hörbar sind.“ (Niethammer 1980: 7).

Auch in der Wissenschaftsgeschichte gewann die Oral History um 1970 an Bedeutung (Doel 2003; te Heesen 2018, 2020, 2022; de Chadarevian 1997). Hatte die Funktion der Wissenschaftsgeschichte bis ins frühe 20. Jahrhundert hinein darin bestanden, an die „großen Männer“ der Wissenschaft und ihre Entdeckungen zu erinnern, so wurde der Glaube an Rationalität und Fortschritt durch die Weltkriege erschüttert (Hagner 2015; te Heesen 2020). Nach den Erfahrungen von industriellem Massenmord und nuklearer Vernichtung ließ sich die Geschichte der modernen Wissenschaft und Technik kaum mehr als teleologische Fortschrittsgeschichte erzählen. Neue Fragen wurden gestellt: Wie wurde dieses Wissen generiert, und wie konnte es seine zerstörerische Kraft entfalten? (te Heesen 2020: 86f.) Die Wissenschaftsgeschichte folgte dem Trend der Sozialgeschichte, indem sie nicht mehr nur „große Männer und ihre Heureka-Momente“ in den Blick nahm, sondern die sozialen, politischen und kulturellen Faktoren der Wissensgenerierung (Keuck et al. 2018: 41; Doel 2003: 357). Dementsprechend entstanden Oral-History-Projekte, die eine Vielzahl von Stimmen – und nicht nur die prominenter Wissenschaftler:innen – hörbar und sichtbar machten.^6^ Zur gleichen Zeit wurde die Wissenschaftsgeschichte vom *practical turn* erfasst, der die Aufmerksamkeit auf die experimentellen Praktiken, Routinen, Kontingenzen und Materialitäten der Wissensproduktion legte (vgl. de Vries & Leezenberg 2019; Rheinberger 2001). Charles Weiner schrieb dazu: „In short, oral history can help us learn more about what it means to ‚do science‘ than is revealed in the public or private written record.“ (Weiner 1988: 548).

Trotz ihres innovativen Potenzials für die Wissenschaftsgeschichte haben kritische Stimmen auf die Grenzen der Oral History hingewiesen – insbesondere was die subjektive, selektive und gegenwartsbezogene Natur der menschlichen Erinnerung betrifft (vgl. de Chadarevian 1997; Müller-Hill 2001; Merchant 2019). Für die Wissenschaftsgeschichte birgt die Interaktion zwischen Historiker:innen und Wissenschaftler:innen, wie Soraya de Chadarevian betont, eine „in-built tension“ (de Chadarevian 1997: 62; Merchant 2019). Einerseits sind Historiker:innen auf das Vertrauen ihrer Gesprächspartner:innen angewiesen, um ihnen teils heikle Informationen zu entlocken; andererseits müssen sie ihre Interviews auf ihr Erkenntnisinteresse hin ausrichten, um die für sie relevanten Informationen zu erhalten. Dieses Spannungsverhältnis kommt dann zum Vorschein, wenn unbequeme Wahrheiten ausgesprochen werden, die womöglich die Reputation eines beziehungsweise einer Wissenschaftler:in oder Institution gefährden (de Chadarevian 1997: 62). Nicht selten geraten Archivquellen mit den Erinnerungen der interviewten Person in Konflikt. Die Physikhistorikerin Lillian Hoddeson unterscheidet in diesem Zusammenhang zwischen der Selbstdarstellung einer Person (*mask*) und dem authentischen Ich (*face*). Durch den geschickten Einsatz von Dokumenten, einer pragmatischen Mischung aus sachlicher Distanz und persönlicher Annäherung, Kollaboration und Konfrontation könne der beziehungsweise die Interviewer:in die Maske ihres Gegenübers lüften und Erinnerungen entlocken, die sich von den eingeübten und tradierten Narrativen lösen (Hoddeson 2006).

Das erste und wohl bekannteste Oral-History-Projekt in der Wissenschaftsgeschichte war das von Thomas Kuhn zwischen 1961 und 1964 geleitete Projekt Sources for History of Quantum Physics (Kuhn et al. 1967). In New York ließ sich Kuhn von Saul Benison (1920–2006), einem Schüler Nevins’, zur Oral History beraten. Auf Basis von Zeitzeug:inneninterviews versuchte Kuhn die epistemischen Schritte nachzuvollziehen, die zu den Durchbrüchen in der Quantenphysik des 20. Jahrhunderts geführt hatten. Sein Projekt fokussierte weniger auf den Begründungszusammenhang wissenschaftlicher Erkenntnisse – wie etwa in der Tradition des Wiener Kreises –, sondern auf ihren Entdeckungszusammenhang. Konzeptionell blieb Kuhn dem Eliteninterview treu: Gezielt suchte er sich die Koryphäen des Fachs als Interviewpartner – angefangen bei James Franck über Niels Bohr bis Werner Heisenberg – und begab sich dazu auf eine „Grand Tour“ der Physik (te Heesen 2022: 16, 84).

In ihrem Buch *Revolutionäre im Interview* (2022) identifiziert Anke te Heesen drei Merkmale der Oral History in den 1960ern: Zum einen gab es noch keine ausformulierte Theorie oder Methode der Oral History, als Kuhn sein Sources-Projekt begann. Als Instrument und Forschungspraxis war das Interview noch im Werden begriffen, wurde in verschiedenen Disziplinen wie der empirischen Sozialforschung und Psychologie weiterentwickelt und wies fließende Übergänge zum Journalismus auf. Zum anderen variierten die Interviews in ihrer Funktion, je nachdem welches Verhältnis der Interviewer und sein Gegenüber hatten: Mal nahmen die Interviews die Gestalt eines Zeitzeug:inneninterviews an, deren O‑Ton ein authentisches Bild der Vergangenheit vermitteln sollte, ein anderes Mal dienten sie schlicht der Beschaffung und Sammlung von Informationen (te Heesen 2022: 173ff.). Zum dritten verdeutlichte Kuhns Sources-Projekt, dass Oral-History-Interviews ein hohes Maß an Selbstreflexion abverlangen, da Historiker:innen durch den Akt des Befragens ihre Quelle miterschaffen (te Heesen 2022: 175; Keuck et al. 2018: 43). Jede Frage gestaltet den Verlauf des Interviews und damit seinen Erkenntnisgewinn mit. Die genannten Merkmale treffen auch auf die Forschungspraxis im Kontext der Recombinant DNA History Collection zu. Dass die Oral-History-Interviews zur Reproduktion bestehender Biases und gar zur Bildung neuer Mythen beitrugen, sehen wir im Folgenden.

## Asilomar und die Folgen: Die Debatte um rekombinante DNA

Dass sich DNA-Moleküle innerhalb einer Spezies neu miteinander verbinden, war in der Biologie seit Längerem bekannt. Das Neuartige am Verfahren der rekombinanten DNA war die Übertragung von Genen zwischen unterschiedlichen Spezies. Zwischen 1971 und 1973 gelang es einem Forscher:innenteam um den Stanforder Molekularbiologen Paul Berg (1926–2023) erstmals, die Chromosomen eines Tumorvirus (SV40) in einen E. coli-Bakterienstamm einzuschleusen (Salem 2013: 63). So vielversprechend die neue Technologie war, um die Funktionsweise von Genen oder die genetischen Ursachen von Krankheiten zu erforschen, so wenig wusste man über mögliche Risiken und Gefahren. Konnten transgene Organismen zur Gefahr für die öffentliche Gesundheit werden? War es ethisch, Gene über die Speziesgrenze hinweg zu transferieren? Und würden die neuen Rekombinationstechniken früher oder später zu einem *Genetic Engineering* am Menschen führen? Offene Fragen, auf die niemand eine sichere Antwort hatte.

Während die Diskussion zunächst auf einen begrenzten Wissenschaftler:innenkreis beschränkt blieb, beschlossen die Teilnehmer:innen der Gordon Conference on Nucleic Acids 1973, mit ihren Bedenken an die Öffentlichkeit zu gehen. Im sogenannten Berg Letter, der 1974 in *Science* und *Nature* erschien, einigten sich führende Molekularbiolog:innen auf ein freiwilliges Moratorium – das heißt den vorläufigen Aufschub weiterer rDNA-Experimente (Berg et al. 1974). Ein halbes Jahr später, im Februar 1975, fand im kalifornischen Asilomar eine groß angelegte Konferenz statt, auf der mögliche Risiken und Gefahren von rDNA-Experimenten diskutiert wurden. Diese ging als Asilomar-Konferenz in die Geschichte der Lebenswissenschaften ein (Berg et al. 1975; Fredrickson 2001; Hurlbut 2015; Jasanoff 2005; Salem 2013; Wright 1994). Genau genommen handelte es sich bei der Konferenz um Asilomar II, denn bereits im Januar 1973 hatte dort ein Treffen zur Erörterung von Sicherheitsfragen bei Experimenten an Tumorviren stattgefunden (Salem 2013: 64). Finanziert wurde die Asilomar-II-Konferenz von der National Science Foundation (NSF), dem National Cancer Institute (NCI) und den National Institutes of Health (NIH) (Salem 2013: 66). Insgesamt 155 Teilnehmer:innen folgten der Einladung, von denen die meisten Molekularbiolog:innen waren, gefolgt von Genetiker:innen, Biochemiker:innen, Virolog:innen und Embryolog:innen. Unter den Teilnehmer:innen befanden sich zudem 16 Journalist:innen sowie drei Juristen und ein Medizinethiker.^7^ Nach drei Tagen intensiver Diskussion kam die Konferenz zu dem Schluss, das Moratorium aufzuheben und Richtlinien (*Guidelines*) zur Regulierung von rDNA-Experimenten zu erarbeiten (Berg et al. 1975). Mehr als ein Jahr nach Asilomar, am 23. Juni 1976, veröffentlichten die NIH ihre Richtlinien zur Regulierung von rDNA-Experimenten, die eine Kombination aus „physical“ und „biological containment“ vorsahen. Die erste Kategorie bezog sich auf physische Barrieren zur Gewährleistung der Laborsicherheit (Schutzkleidung, Luftschleusen etc.), wohingegen sich die zweite Kategorie auf Modellorganismen bezog, die außerhalb der Laborbedingungen nicht überleben konnten (Salem 2013: 67f.).

Obwohl die Organisator:innen von Asilomar gehofft hatten, die Risikofrage auf eine innerwissenschaftliche Auseinandersetzung eingrenzen zu können, wuchs sie rasch zu einer öffentlichen Debatte heran, in die sich Stimmen aus Wissenschaft, Politik, Presse und Oppositionsgruppen einmischten (Altimore 1982; Botelho 2019; Krimsky 1982; Wright 1994). Selbst der *Rolling Stone* berichtete im Juni 1975 in einer mehrseitigen Reportage über den „Pandora’s Box Congress“ (Rogers 1975). In verschiedenen US-amerikanischen Unistädten kam es zu heftigen Auseinandersetzungen um die Frage, ob riskante rDNA-Experimente vor Ort durchgeführt werden sollten. Die wohl bekannteste Kontroverse entbrannte in Cambridge, Massachusetts im Sommer 1976, die als „Cambridge controversy“, „Cambridge episode“ oder „Cambridge debate“ in die Geschichte einging (vgl. Botelho 2019; Krimsky 1982; Watson & Tooze 1981; Wright 1994: 222). Was war geschehen? Im Frühjahr 1976 wurde bekannt, dass das Molecular Biology Department an der Harvard University den Plan verfolgte, zwei Räume in ein P3-Labor (*High Containment Facility*) für riskante Gentechnikversuche auszubauen. Das Bauvorhaben, für das die NIH 500.000 US-Dollar bereitstellten, ging auf die Initiative des Molekularbiologen Mark Ptashne zurück (Cobb 2022: 92).^8^ Als das Vorhaben fakultätsintern besprochen wurde, reagierten einige Biolog:innen in Harvard mit Sorge und Protest – allen voran Ruth Hubbard (1924–2016) und ihr Ehemann, der Physiologe und Nobelpreisträger George Wald (1906–1997).^9^

Nachdem der damalige Bürgermeister von Cambridge, Alfred Vellucci, durch einen Artikel im *Boston Phoenix* von den Laborplänen erfahren hatte, regte er – nach Gesprächen mit Hubbard und Wald – eine öffentliche Anhörung im Stadtrat von Cambridge an (Gottlieb & Jerome 1976). Aus einer italienisch-US-amerikanischen Arbeiterfamilie stammend, war Vellucci berüchtigt für seine kritische Haltung gegenüber Harvard. Schon vor dem Eklat um das P3-Labor war Vellucci mit der Harvard University aufgrund von Steuerprivilegien, welche die Universität traditionell genoss, in Konflikt geraten.^10^

Am 23. Juni 1976 – dem Tag der Veröffentlichung der NIH-Guidelines – fand die erste öffentliche Anhörung vor dem Stadtrat von Cambridge statt, auf der Bürgermeister Vellucci seinen „flair for the dramatic“ unter Beweis stellte (Mendelsohn 1984: 324). Verschiedene Biolog:innen – unter ihnen Mark Ptashne, Maxine Singer, Ruth Hubbard und Jonathan King – gaben ein öffentliches Statement ab und stellten sich den teils provokanten Fragen Velluccis und anderer Mitglieder des Stadtrats. Aus Angst, Vellucci werde die rDNA-Forschung in Cambridge vollends verbieten, wandten sich die Nobelpreisträger James Watson und Paul Berg per Brief an den Bürgermeister.^11^ Doch die Schlichtungsversuche von Watson und Berg schienen bei Vellucci auf taube Ohren zu stoßen. Nach einer zweiten Anhörung am 7. Juli verabschiedete der Stadtrat von Cambridge ein halbjähriges Moratorium auf sämtliche rDNA-Experimente. Ergänzend dazu wurde die Gründung eines Komitees aus Bürgervertreter:innen beschlossen, das Risiken vor Ort einschätzen und Empfehlungen zum Umgang mit rekombinanter Forschung in Cambridge abgeben sollte (Krimsky 1982; Botelho 2019: 303). Das neu gegründete Cambridge Experimentation Review Board (CERB) bestand aus neun Bürgervertreter:innen, darunter der Wissenschaftsphilosoph und Science-Policy-Experte Sheldon Krimsky (zu CERB vgl. Waddell 1989, 1990).^12^ Das Komitee traf sich fünf Monate lang und nahm Expert:innen zum Pro und Kontra der Gentechnik ins Kreuzverhör.

Mark Ptashne erhielt schließlich grünes Licht für den Bau des P3-Labors: Am 5. Januar 1977 gab das CERB in Abstimmung mit dem Bürgermeister seine Empfehlungen zum geplanten Labor ab, die in einer neuen Stadtverordnung (*Biosafety Ordinance*) mündeten. Diese bildete die erste Gesetzgebung in den USA zur Regulierung der rDNA-Forschung. Im Wesentlichen folgte die Verordnung den NIH-Guidelines, allerdings wurden zusätzliche Sicherheitsvorkehrungen und die Einrichtung eines Biohazards Committee beschlossen, das die Einhaltung der NIH-Guidelines vor Ort beobachten sollte (Krimsky 1982: 307ff.). Auch in anderen US-amerikanischen Städten kam es zu Auseinandersetzungen um rDNA-Forschung, die in lokalen Verordnungen mündeten (San Diego, Princeton, Berkeley u. a.). Die wohl größte Wirkung hatte die Cambridge-Kontroverse auf die Diskussionen in Washington, D.C.: Senator Edward Kennedy war es zu verdanken, dass die rDNA-Frage vom Kongress auf die Tagesordnung gesetzt wurde (Wright 1994: 222). Zwischen 1976 und 1978 debattierte der Kongress über mehr als ein Dutzend Gesetzesvorlagen zur Regulierung der rekombinanten Forschung auf Bundesebene, doch eine Einigung konnte nicht erzielt werden (Weiner 1999: 297).

Die Affäre um das Harvard-Labor hätte ironischer nicht ausgehen können: Zum einen wurde das neu gebaute Labor nie für P3-Experimente genutzt, da die NIH-Guidelines zum Zeitpunkt der Fertigstellung bereits gelockert worden waren und Ptashne seine Experimente in einem gewöhnlichen Laborsetting durchführen konnte (Cobb 2022: 97). Zum anderen: Als im Jahr 1983 Biogen als erstes Biotech-Unternehmen in Cambridge seine Pforten öffnete, betonte kein geringerer als Alfred Vellucci bei seiner Eröffnungsrede, dass er keine Sorge vor rekombinanter Forschung in Cambridge habe, solange diese „Steuern“ zahle (Durant 2012: 405).

## History in the Making: Die Recombinant DNA History Collection am MIT

Wenige Monate nach der Asilomar-Konferenz im Frühjahr 1975 wurde am MIT eine neue Oral-History-Sammlung unter der Federführung von Charles Weiner angelegt: Die Recombinant DNA History Collection. Eine Auflistung von Ronald E. Doel zeigt, dass diese Sammlung eine der ersten ihrer Art in den USA und Kanada war.^13^ Neben dem MIT schufen das Smithsonian, die Columbia University, das American Institute of Physics, die NASA und das Institute of Electrical and Electronics Engineers (IEEE) in den 1960er und 1970er Jahren ihre eigenen Oral-History-Sammlungen. Später folgten das Cold Spring Harbor Laboratory und das Science History Institute dem Beispiel ihrer Vorgänger (Doel 2003: 352).

Charles Weiner hatte am Case Institute of Technology Metallurgie studiert und wechselte nach seinem Bachelor-Abschluss zur Wissenschaftsgeschichte, worin er 1965 promoviert wurde. Von 1965 bis 1974 war er Direktor des Center for the History of Physics und der Niels Bohr Library am American Institute of Physics, wo er umfangreiche Erfahrungen mit Zeitzeug:inneninterviews zur Geschichte der Atomphysik sammelte. Seine Oral-History-Interviews, Quellen und Forschungen zur Physikgeschichte des 20. Jahrhunderts flossen in vier Bände ein, die er mitherausgab: *Exploring the History of Nuclear Physics* (1972), *The Legacy of George Ellery Hale: Evolution of Astronomy and Scientific Institutions* (1972), *History of Twentieth Century Physics* (1977) und *Robert Oppenheimer: Letters and Recollections* (1980). Im Jahr 1975 wechselte er zum MIT, wo er die Leitung der Oral-History-Abteilung innerhalb des Science and Society-Programms übernahm. In dieser Funktion legte Weiner seinen Schwerpunkt auf die ethischen, sozialen und politischen Folgen von Wissenschaft, insbesondere der Nuklearphysik und Biotechnologie. Über zwanzig Jahre arbeitete er an einem Buch zur Geschichte der sozialen Verantwortung von Wissenschaftler:innen von der Atombombe bis zum Gentechnikzeitalter, das allerdings unvollendet blieb.^14^ Im Jahr 2002 hatte Weiner die Ehre, am MIT die jährliche „Arthur Miller Lecture on Science and Ethics“ zu halten. Zeit seines Lebens, so legen es mehrere Nachrufe nahe, blieb Weiner linken, sozialistischen Ideen eng verbunden (Olwell et al. 2014).

Weiner erkannte früh die historische Bedeutung der rDNA-Debatte für Wissenschaft, Öffentlichkeit und Politik. Der Molekularbiologe David Baltimore machte ihn auf das Thema aufmerksam, als er am 6. November 1974 am MIT den fakultätsinternen Vortrag „Where does molecular biology become more of a hazard than a promise?“ vor einem interdisziplinären Publikum hielt (Baltimore 1974). In seinem Vortrag stellte Baltimore die neuen Rekombinationstechniken vor, reflektierte über mögliche Gefahren und sprach über die geplante Asilomar-Konferenz. Wie ein roter Faden durchzog Baltimores Vortrag ein moralisches Dilemma: Obwohl er und seine Kolleg:innen darum bemüht waren, verantwortungsvoll mit der neuen Technologie umzugehen, hatten sie Angst vor einer zu strikten staatlichen Regulierung. „We’re stuck between self-determination of limits and imposition of orthodoxy. We’re stuck between self-interest of scientists and the public interest“, konstatierte er (ebd.). Weiner erinnerte sich: „As I listened I was impressed with this effort for responsibility and self-regulation, but I wondered how it was possible to *exclude* the public in a matter that *should* be of public concern“ (Weiner 2001: 208). Diese Fragen, so Weiner, weckten seine Neugier als Wissenschaftshistoriker, denn seit Längerem interessierte er sich für die Entwicklung neuer Forschungsfelder und die Weise, wie Wissenschaftler:innen ihre soziale Verantwortung wahrnahmen. Die Tonbandaufnahmen dieses Meetings bildeten den Auftakt eines Oral-History-Projekts, das zu einer ganzen Archivsammlung heranwachsen sollte (Weiner 1979b: 281).

Die Recombinant DNA History Collection war das Herzstück der Technology Studies am MIT, die 1974 von dem Sinologen und Wissenschaftshistoriker Nathan Sivin (1931–2022) neu eingerichtet wurden. Sie waren die Vorgänger des heutigen Science, Technology and Society-Programms am MIT. Ziel des interdisziplinären Programms war es, Bachelorstudierenden (*undergraduates*) ein kritisches Bewusstsein über die gesellschaftlichen Voraussetzungen und Implikationen ihres zukünftigen Handelns in Wissenschaft und Technologie zu vermitteln.^15^ Das Programm sollte Forschende unterschiedlicher Fächer – angefangen von der Anthropologie, Archäologie, Geschichts- und Politikwissenschaft über die Physik, Ingenieurswissenschaft und Raumfahrtforschung bis zur Architektur und Materialwissenschaft – zusammenbringen. Die Oral History wurde dabei als Baustein einer innovativen Lehre angesehen. Im Exposé für das Technology Studies-Programm, das der Fakultät vorgelegt wurde, hieß es:

„Use of oral history techniques by our students is one example of a new kind of learning experience which the Program provides. The Program will continue to experiment with teaching situations which go beyond traditional classroom settings. Opportunities for field work, research by participant observation and student internships in science and engineering will be investigated as ways of enlarging the scope of students work in Technology Studies.“^16^

Aus der Korrespondenz zwischen William Speer und Nathan Sivin geht hervor, dass die technische Ausstattung des Oral-History-Programms für damalige Verhältnisse durchaus üppig war.^17^ Zudem holte sich Sivin Rat von anderen Wissenschaftshistoriker:innen, was die Umsetzung und Integration der Oral History in die Technology Studies betraf. Der Wissenschaftshistoriker Daniel Kevles warnte in einem Brief vom 14. März 1974 vor methodischen Fallstricken, die Oral-History-Interviews mit sich brachten: „When interviews are conducted merely for the sake of conducting interviews, the result is more often than not some useful information and a great many lost opportunities. In short, I would urge that for maximum effectiveness you construct your oral history program around active scholars.“^18^ Parallel zur Recombinant DNA History Collection entstand am MIT eine zweite Sammlung, die sich auf autobiografische Interviews von Wissenschaftlerinnen aus den Lebens‑, Natur- und Ingenieurswissenschaften spezialisierte: The Women in Science and Engineering Collection (Weiner 1988: 556ff.).

Weiners Oral-History-Projekt dauerte vier Jahre (1975–1979) und wurde unter anderem aus Mitteln der National Science Foundation und des National Endowment for the Humanities finanziert (Weiner 1978). Die Sammlung richtete sich sowohl an Studierende und Lehrende der Fakultät als auch an Historiker:innen, die zur Geschichte der Gentechnikdebatte forschten. Der Schwerpunkt der Sammlung lag erstens auf Interviews mit Lebenswissenschaftler:innen, die an rDNA forschten; mit politischen Entscheidungsträger:innen, die Richtlinien und Gesetze erarbeiteten; mit Aktivist:innen und öffentlichen Kritiker:innen der rDNA und mit Journalist:innen, die über die rDNA-Debatte berichteten (Weiner 1979a: 17). Zweitens umfasste die Sammlung rund 36.000 Seiten Schriftgut, darunter Briefkorrespondenzen, Presseausschnitte, Vorträge, Protokolle, Konferenzbeiträge und Gesetzestexte. Ein dritter Sammlungsschwerpunkt lag auf Video- und Tonaufnahmen von Anhörungen, Pressemitteilungen, Stellungnahmen, Vorträgen und Meetings, vor allem während der Cambridge-Kontroverse (Weiner 1978, 1979a). Um seiner Sammlung mehr Sichtbarkeit zu verleihen, veröffentlichte Weiner 1978/79 kurze Projektberichte im US-amerikanischen STS-Journal *Science, Technology & Human Values* (Weiner 1978, 1979a).^19^ Die Sammlung trug rasch erste Früchte: Bereits 1979, vier Jahre nach Projektbeginn, hatten mehr als 80 Personen aus verschiedenen Institutionen der USA die Sammlung für Studien- und Forschungszwecke genutzt (Weiner 1979a: 18f.).

Unterstützung erhielt Weiner von der Kommunikationswissenschaftlerin Rae Goodell, die in ihrer Dissertation *The Visible Scientist* (1977) untersuchte, welche Strategien Wissenschaftler im Umgang mit Medien und Öffentlichkeit anwendeten, um politischen Einfluss zu gewinnen. Goodell beschrieb in ihrer Arbeit einen neuen Typus Wissenschaftler im massenmedialen Zeitalter, den sie als „visible scientist“ klassifizierte. Die Sichtbarkeit von Wissenschaftlern, so ihr Argument, resultierte weniger aus ihren spektakulären Entdeckungen und deren Popularisierung, sondern aus ihrem geschickten Umgang mit den Massenmedien, den sie bei öffentlichen Kontroversen einsetzten (vgl. Goodell 1977). Exemplarisch untersuchte sie hierfür *Public Intellectuals* wie Paul Ehrlich, Noam Chomsky, James Watson oder Burrhus F. Skinner. Mit *The Visible Scientist* schuf Goodell ein Konzept, das methodisch wie theoretisch großen Einfluss auf das damals noch junge Feld *Public Understanding of Science* hatte; ihre Arbeit regte weitere Forschungen zur Rolle von öffentlicher Kommunikation im Wissenschaftssystem und zum Einfluss der Medien auf den Prozess der Wissensproduktion an (Fahy 2017: 1019–1024).

Goodell schien eine ideale Kooperationspartnerin für Weiners Oral-History-Projekt zu sein, denn für ihr Dissertationsprojekt führte sie mit mehr als vierzig Wissenschaftlern narrative Interviews. Innerhalb von vier Jahren sammelten, verzeichneten und katalogisierten Weiner und sein Team circa 5.000 Dokumente rund um die rDNA-Debatte, zeichneten Konferenzen, Meetings und Anhörungen auf lokaler und nationaler Ebene auf und führten Interviews mit 122 Akteur:innen der rDNA-Debatte, darunter mit 36 Teilnehmer:innen der Asilomar-Konferenz.^20^ Aus einem Brief von Rae Goodell an den Molekulargenetiker und Nobelpreisträger Joshua Lederberg (1925–2008) geht hervor, dass Weiner und Goodell an einem gemeinsamen Buch, einer „documentary history of recombinant DNA developments“, arbeiteten – ein Projekt, das jedoch nie verwirklicht wurde.^21^

Gemäß einer „history in the making“ hatte Weiners Oral-History-Sammlung zum Ziel, Wissenschaftsgeschichte im Werden zu dokumentieren (vgl. Dorman 1980; Weiner 1988). In einem späteren Aufsatz appellierte Weiner an Wissenschaftshistoriker:innen, nicht nur die Vergangenheit, sondern auch die geschichtemachenden Ereignisse der Gegenwart stärker in den Blick zu nehmen: „We are so busy catching up with the past (an impossible task) that we neglect the history in which we are embedded and overlook events ocurring around us“ (Weiner 1988: 559). Weiner blickte gewissermaßen mit den Augen eines zukünftigen Historikers auf die neue, noch im Werden begriffene Technologie. Ihn interessierten all jene Dokumente, Stimmen und Ereignisse, die er für eine zukünftige, noch zu schreibende Geschichte der Biotechnologie für relevant erachtete. In Vorwegnahme einer noch zu schreibenden Geschichte hielt er diese Stimmen dokumentarisch fest, verzeichnete und archivierte sie: „Historians can play important roles as witnesses to contemporary events, creating a contemporary record in anticipation of its value for the future. Why wait thirty or forty years to interview someone?“ (Weiner 1988: 559). Wie aus diesem Zitat hervorgeht, attestierte Weiner Historiker:innen eine spezifische Rolle als Zeitzeug:innen: Sie bestand im sensorischen Gespür für die historische Bedeutung gegenwärtiger Ereignisse, die es für die Nachwelt zu speichern galt.

Obwohl Weiner seine Sammlung als „unique experiment in documenting the contemporary history of science“ bezeichnete, war er keineswegs der erste, der die Oral History als Methode für die Wissenschaftsgeschichte entdeckte (Weiner 1979a: 17). Wie schon erwähnt, leitete Thomas Kuhn zwischen 1961 und 1964 unter dem Titel Sources for History of Quantum Physics ein umfangreiches Oral-History-Projekt, in dem die Koryphäen der Quantenphysik zur Genese ihrer Entdeckungen befragt wurden (te Heesen 2020, 2021, 2022). Kuhn, der gerade an seinem Opus Magnum *Structures of Scientific Revolutions* (1962) arbeitete, versuchte mit seinem Interviewprojekt eine empirische Fundierung für seine These zu finden, dass wissenschaftliche Revolutionen einer strukturellen Dynamik von Paradigmenwechseln folgten (te Heesen 2022: 14). Was als ambitioniertes Projekt begann, entpuppte sich jedoch bald als herbe Enttäuschung, denn die interviewten Physiker waren nicht gerade die „besten Zeugen ihrer eigenen Vergangenheit“ (te Heesen 2022: 170). Trotz enttäuschter Erwartungen ebnete Kuhn mit seinem Sources-Projekt den Weg für eine neue Wissenschaftsgeschichte, die sich von alten Hagiografien, dem logischen Positivismus, der Ideengeschichte und dem dogmatischen Gegensatz von Internalismus und Externalismus emanzipierte (te Heesen 2022: 170–173).

Für die Historisierung der rDNA-Debatte war die Oral-History-Sammlung am MIT von zentraler Bedeutung. Sämtliche Arbeiten zur Gentechnikdebatte in den USA und Europa – etwa Sheldon Krimskys *Genetic Alchemy* (1982), Susan Wrights *Molecular Politics* (1994) oder Herbert Gottweis’ *Governing Molecules* (1998) – bezogen einen wesentlichen Teil ihrer Quellen aus der Recombinant DNA History Collection. Da Sheldon Krimsky selbst Mitglied im Bürgerkomitee CERB war, warfen ihm schon damalige Rezensent:innen von *Genetic Alchemy* einen „bias“ vor (Newell 1984; Setlow 1985). Nicht zuletzt war es Weiners Sammlung mit zu verdanken, dass die Cambridge-Kontroverse zum Präzedenzfall für „public participation“ in wissenschaftspolitischen Entscheidungen wurde (vgl. Botelho 2019; Durant 2012; Feldman & Lowe 2008; Krimsky 1982; Mendelsohn 1984; Nelkin 1978a, b). Wie wir im Folgenden sehen, verfolgte Weiner mit seiner Oral-History-Sammlung das Ziel, alte Wissenschaftsmythen zu widerlegen.

## Alte Mythen widerlegen: Oral Histories der Gentechnikdebatte

### Oral History Interviews

Charles Weiner attestierte dem Oral-History-Interview für die moderne Wissenschaftsgeschichte eine aufklärerische und damit auch politische Funktion. Ihr subversives Potenzial bestand darin, alte Wissenschaftsmythen herauszufordern und zu einem neuen, kritischen Wissenschaftsverständnis beizutragen. In dem Aufsatz „Oral History of Science: A Mushrooming Cloud?“, der 1988 im *Journal of American History* erschien, betonte Weiner: „Used with other sources, oral history can help to penetrate the mystique of science as a neutral, value-free enterprise solely concerned with the disinterested search for truth about the natural world through the application of rational methodology.“ (Weiner 1988: 549) Durch die Einbeziehung diverser Stimmen sollte die Oral History den Mythos widerlegen, Wissenschaft sei ein neutrales, objektives und wertfreies Unterfangen, das allein und ausschließlich dem Erkenntnisfortschritt diene. Bereits in seiner Beschäftigung mit der Nuklearphysik und dem Manhattan-Projekt zwischen 1965 und 1974 gelangte Weiner zu der Einsicht, dass Wissenschaft und Politik nicht voneinander zu trennen seien – so hatte er etwa die Briefe Robert Oppenheimers herausgegeben und Interviews mit Richard Feynman geführt (Weiner 1977; Kimball Smith & Weiner 1980). Ebenso bewegte sich Weiner wie auch andere Kolleg:innen des MIT und der Harvard University im intellektuellen Mikrokosmos der Boston Area, der durch herrschaftskritische Diskurse der Neuen Linken geprägt war (Moore 2008; Van Gosse 2005). Ausgehend von den Anti-Vietnam-Protesten kritisierten linke Gruppen wie Science for the People den „military-industrial complex“ und die „Jason scientists“, polemisierten gegen die „scientific elite“ und forderten stattdessen eine humane, demokratische Wissenschaft (Moore 2008; King 1977; Botelho 2019).^22^ Science for the People hatte in Boston eine ihrer aktivsten Ortsgruppen, die in der Gentechnik und Soziobiologie Edward O. Wilsons die Gefahr eines „biologischen Determinismus“ sah – die Übertragung evolutionsgenetischer Deutungsmuster auf sämtliche Aspekte des Menschen: seine Intelligenz, sein Verhalten, seine Kultur und gesellschaftliche Organisation (Segerstråle 2001; Stuhrmann 2023; Weidman 2021).^23^

Auf einer Konferenz zum Thema „Recombinant DNA and Genetic Experimentation“ im britischen Kent, die im April 1979 vom Committee of Genetic Experimentation (COGENE) und der Royal Society veranstaltet wurde, hielt Weiner einen Vortrag über den historischen Verlauf der rDNA-Kontroverse. Dabei nutzte er auch die Gelegenheit, die vielzitierte Trennung von Wissenschaft und Politik als naiven Irrglauben zu entlarven:

„I disagree with the statement made by some biologists that now they are acting scientifically when they maintain that the conjectured risks were overstated, and that they were acting politically five years ago when they voiced concern about such risks. Such statements are bound to be seen by the public as self-serving. In any case, it is naïve to think that science and politics can be neatly separated. Science is imbedded in a social context, and recombinant DNA, like other fields, will continue to be subject to pressures and questioning, especially as it becomes more successful and has greater impact on society.“ (Weiner 1979b: 287)

In einem Interview mit Janis Dorman verglich Weiner seine Oral-History-Methode mit den Village Studies in der Ethnologie; jenen Studien, die durch teilnehmende Beobachtung die soziale Ordnung afrikanischer Dörfer zu erforschen versuchten. Der scharfe Beobachter, so Weiner, könne in den Interviews herausfinden, wie die Befragten ausgebildet wurden, welche Werte sie vertraten und wie diese geformt wurden:

„The interviews were not opinion polls. They were to get first person testimony of what they understood, what they perceived, what they did, in great detail. We also tried to get a great deal of background information from the individuals about their prior concerns for these issues, their research backgrounds, their training. We tried to get an understanding of how their personal values were formed; where their concepts of ethical issues were formed.“ (Dorman 1980: 87)

Weiner versuchte durch seine Interviews zu zeigen, wie sich im Zuge der Debatte die Wahrnehmung potenzieller Gefahren innerhalb der Scientific Community veränderte und wie Wissenschaftler:innen auf den Druck der Öffentlichkeit reagierten (Weiner 1988: 557). Er war sich bewusst, dass die meisten Oral Histories die Stimmen der weißen, männlichen Elite einfingen. Um ein multiperspektivisches Bild dieser Kontroverse zu erhalten, müssten Historiker auch die Perspektiven von Frauen, Lai:innen, Veteran:innen und marginalisierten Gruppen berücksichtigen, so Weiner. „Historians must read the letters and hear the stories of atomic veterans, as well as atomic scientists“ (Weiner 1988: 558). Obwohl Weiner die Oral History für eine wertvolle, häufig unterschätzte Quelle in der Wissenschaftsgeschichte hielt, war er sich auch ihrer Grenzen bewusst. Wiederholt erinnerte er daran, dass Oral-History-Interviews nur dann gewinnbringend genutzt werden könnten, wenn sie in Kombination mit schriftlichen Quellen, Archivalien, Ton- und Bildmaterial ausgewertet wurden. Denn Erinnerungen – und das galt schon damals als Konsens in der Geschichtswissenschaft – waren subjektive, selektive und sozial bedingte Rekonstruktionen der Vergangenheit, die zahlreiche Fallstricke bargen (Dorman 1980: 87f.).

Weiner und Goodell führten über 120 Interviews, die auf Audioband aufgenommen, im Anschluss transkribiert, mit einem Sach- und Personenverzeichnis versehen und von den Interviewpartner:innen freigegeben wurden. Die Interviews folgten keiner festen, vorgegebenen Struktur, sondern variierten je nach Person, die befragt wurde. Natürlich machte es einen Unterschied, ob der Molekularbiologe und rDNA-Pionier Paul Berg oder die Wissenschaftshistorikerin und Science for the People-Aktivistin Susan Wright zu potenziellen Gefahren und politischen Folgen der Gentechnik interviewt wurde. Dennoch lassen sich gewisse Abläufe der Interviewführung beobachten: Die meisten Interviews begannen mit Fragen zum familiären Hintergrund und zum beruflichen wie akademischen Werdegang. Die anschließenden Fragen zielten darauf ab, wie die Befragten mit dem Thema rDNA in Berührung kamen, wie sie deren Risiken einschätzten und wie sie sich zu Asilomar, Cambridge und den NIH-Guidelines positionierten. In einem Interview mit dem britischen Biologen und damaligen EMBO-Direktor John Tooze ergänzte Weiner seine Frage, ob die Regulierungsmaßnahmen für rDNA-Forschung ausreichend seien, mit folgenden Worten: „That’s just the point of the question: that the immediate issue is safety; the larger issue is something that has a great deal to do with our view of the world and its future, and about cultures and so forth, and about politics.“ (Tooze 1976)^24^

In vielen Interviews nahm die Cambridge-Kontroverse großen Raum ein, da in ihr die politische Natur von Wissenschaft zum Vorschein kam. Das Interview mit der Harvard-Biologin Ruth Hubbard, eine Wegbereiterin der Feminist Science Studies, verdeutlicht, wie sehr Weiner um eine akribische Rekonstruktion der Cambridge-Ereignisse bemüht war (Hubbard 1978). Kleinteilig versuchte er nachzuvollziehen, wer wann etwas von den Laborplänen wusste, wann und wo interne Meetings stattfanden und wie es zu den öffentlichen Anhörungen vor dem Stadtrat in Cambridge kam. Immer wieder hoben seine Fragen auf Hubbards Wahrnehmung der Kontroverse ab, vor allem ihre Einschätzung der öffentlichen Anhörung, wo sie als Expertin befragt wurde. Hubbard widersprach den Vorwürfen der P3-Befürworter:innen, bei der Anhörung habe es sich um einen albernen Zirkus gehandelt. Sie betrachtete die Anhörungen als Versuch, kontroverse Wissenschaftsthemen im öffentlichen Raum auszuhandeln:

„I was fairly excited by the hearings just as a way of getting science out into a public forum. I thought it was pretty interesting. I mean, I know that the proponents of the work felt that it was disgraceful and a circus and so on. I didn’t feel it was either disgraceful or a circus. I thought that it was a fairly genuine effort on the part of some people who had never heard about this before to try to grapple with it. And the fact that some of them couldn’t pronounce ‚recombinant DNA‘ really did not worry me very much.“^25^

Für Weiner bedeutete historische Aufklärung auch Selbstermächtigung, Partizipation und Intervention. In dem Interview mit Janis Dorman betonte er, dass seine Oral-History-Sammlung Zugang zu Wissen biete, mit dem auch Laien an der Debatte partizipieren könnten: „It [the Oral History Collection, C.L.] also gives strength to individuals who are increasingly concerned about the effects of science and technology on their lives and who feel left out of the process. This gives them an opportunity to study the process which would enhance their possibilities for participation in the process; that is a very strong commitment that I have.“ (Dorman 1980: 88) Aus den gesammelten Dokumenten und Oral-History-Interviews zog Weiner Jahre später eine zentrale Schlussfolgerung: Die betroffenen Molekularbiolog:innen seien in hohem Maße besorgt um die öffentliche Meinung gewesen und versuchten diese zu antizipieren, zu kontrollieren, zu beeinflussen (Weiner 2001: 209). Weiner fand in den Interviews also eine weitere Bestätigung für die These, dass Wissenschaftler:innen alles andere als neutrale Erkenntnissuchende sind.

Obwohl Weiner in mehreren Aufsätzen über die Bedeutung der Oral History für die Wissenschaftsgeschichte schrieb, reflektierte er erstaunlich wenig über methodische Grenzen und Herausforderungen. Eine besondere Herausforderung in Weiners Oral-History-Interviews war die Frage der *doppelten Zeug:innenschaft*: Einerseits wurden Zeitzeug:innen zu ihren subjektiven Erinnerungen und Wahrnehmungen der Gentechnikkontroverse befragt, andererseits war Weiner selbst Zeitzeuge der Debatte, der sein ganz eigenes Erkenntnisinteresse im Hinterkopf hatte. Die Interviews sollten seine These untermauern, dass Wissenschaft und Politik nicht voneinander zu trennen seien. Weiner traf auf ähnliche methodische Herausforderungen wie Thomas Kuhn in seinem Sources-Projekt. Sie bestanden darin, dass der Interviewer mit seinen lebendigen Quellen in Interaktion trat, aktiv an der Quellenkonstruktion beteiligt war und dieses Involviertsein auch reflektieren musste (te Heesen 2022: 83). Neben dem Interview griff Weiner auf filmische Mittel zurück, um die Kontroverse zu dokumentieren.

### Die Dokumentation „Hypothetical Risk“

**Abb. 1:**
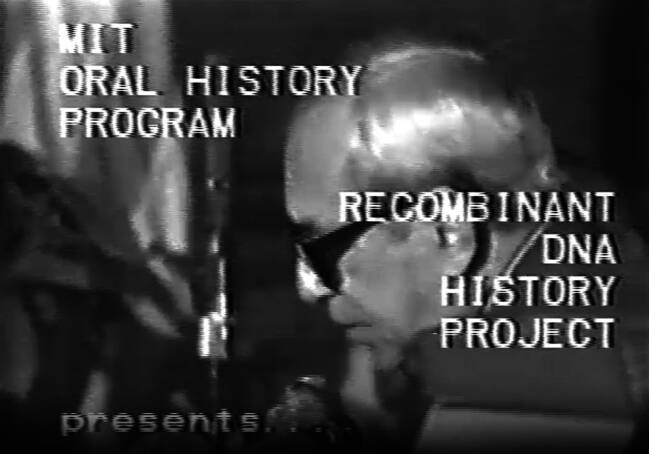
Eröffnungsszene der Dokumentation *Hypothetical Risk* (1977). Zu sehen ist Alfred Vellucci, der damalige Bürgermeister von Cambridge/Massachusetts

Neben Zeitzeug:inneninterviews produzierten Charles Weiner und sein Team auch Video- und Tonbandaufnahmen zur rDNA-Debatte. Das Kernstück seiner Oral-History-Sammlung waren die Videoaufnahmen der öffentlichen Anhörung vor dem Stadtrat von Cambridge am 23. Juni 1976, jenem Tag, an dem die NIH-Guidelines veröffentlicht wurden (Cambridge City Council 1976). Seit 1978 standen die Aufnahmen als halbstündiger Dokumentarfilm mit dem Titel *Hypothetical Risk: Cambridge City Council’s Hearings on DNA Experimentation in Cambridge* in der Recombinant DNA History Collection für Lehr- und Forschungszwecke zur Verfügung (Abb. 1 und 2).^26^ Der Film ist keinem klaren Genre zuzuordnen; vielmehr siedelt er sich an der Schnittstelle von Dokumentation und Lehrfilm an. Weiner nahm die öffentlichen Anhörungen gemeinsam mit Studierenden des MIT auf, die am Recombinant DNA History Project teilnahmen. Für die Aufzeichnung verwendete Weiner einen tragbaren Reel-to-Reel Tape Recorder, der Schwarz-Weiß-Bilder produzierte.^27^

**Abb. 2:**
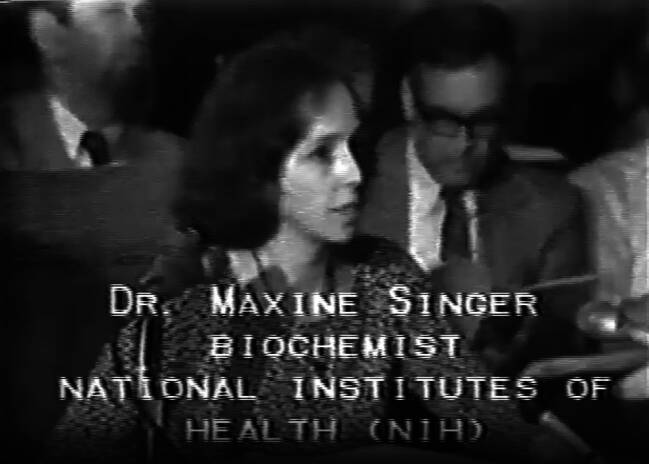
Maxine Singer als Vertreterin der NIH während der Anhörung vor dem Cambridge City Council 1976

In der etwa halbstündigen Videoaufnahme sieht man Ausschnitte der Stellungnahmen verschiedener Biolog:innen, die für und gegen das geplante P3-Labor argumentieren. Neben Bürgermeister Alfred Vellucci stellten auch die Mitglieder des Stadtrats Fragen. Ähnlich wie die Oral-History-Interviews hatte *Hypothetical Risk* nicht nur eine dokumentarische, sondern auch eine didaktische und aufklärerische Funktion: Der Film demonstrierte den Studierenden, dass Wissenschaftler:innen nicht getrennt von Politik und Gesellschaft agierten und widerlegte damit das Bild einer objektiven, neutralen und wertfreien Wissenschaft.

Die Anhörung fand am 23. Juni 1976 um 19 Uhr statt und dauerte laut Überlieferung bis Mitternacht (Durant 2012). Der Saal war bis auf den letzten Platz gefüllt, sogar auf den oberen Rängen. Einige Wissenschaftler:innen, wie etwa der Genetiker David Botstein oder die Wissenschaftshistorikerin Susan Wright, kamen aus anderen Staaten angereist, um der Anhörung beizuwohnen (Feldman & Lowe 2008: 399). Im Publikum protestierten Gegner des P3-Labors, darunter auch Mitglieder von Science for the People, mit Plakaten wie „Build Wisdom Not Containment“ und „No Recombination without Representation“.^28^ Vellucci hatte vorab bewusst den Kontakt zur Presse gesucht, um dem Harvard-Konflikt mehr Sichtbarkeit zu verleihen und sich politisch als „Stimme des Volkes“ zu inszenieren (s. unten). Daher verwundert es kaum, dass am Abend des 23. Juni auch Kamerateams, Journalist:innen und Pressevertreter:innen anwesend waren. In seiner Presseankündigung machte Vellucci keinen Hehl aus seiner ablehnenden Haltung gegenüber den Laborplänen: „I will keep an open mind at this public hearing, but I am not afraid to say that as of now, I am against this project. When you combine genes in a new way, you have no sure way of knowing what will come out of the experiment.“^29^

Vermutlich setzte Vellucci das P3-Labor aus strategischen Gründen auf seine politische Agenda, um seine Beliebtheit in der Bevölkerung zu steigern. Bis auf die beiden Ivy-League-Universitäten war Cambridge in den 1970er Jahren nämlich überwiegend eine *Blue Collar City* – also eine Stadt mit einem traditionellen Arbeitermilieu. Vellucci wollte als Politiker gesehen werden, der sich für die Interessen der „einfachen Leute“ einsetzte. Die Journalistin Barbara Culliton schrieb dazu in *Science*: „With something as esoteric as recombinant DNA, Vellucci had an ideal opportunity to go after Harvard (which has far poorer relations with the city than MIT) while protecting innocent women and children from the menaces of science.“ (Culliton 1976: 300)^30^ Mit diesem Thema versuchte Vellucci offenbar auch die Stimmen des Stadtrats für sich zu gewinnen, die für seine Wiederwahl als Bürgermeister relevant waren (vgl. Goodell 1979).

*Hypothetical Risk* beginnt mit einer Szene, die an eine Gerichtsverhandlung erinnert: Zur Rechten thront Alfred Vellucci wie ein Richter hinter seinem Rednerpult und eröffnet die Verhandlung. Ihm gegenüber sitzen die „Angeklagten“ – der Biochemiker und Molekularbiologe Mark Ptashne, auf den die Planung des P3-Labors zurückgeht, Daniel Branton, Vorsitzender des Harvard Biosafety Committee, sowie Maxine Singer, die die soeben verabschiedeten NIH-Guidelines vorstellt. Vellucci nutzt die Gelegenheit, um seine Rolle als Laie und „Stimme des Volkes“ zu unterstreichen. Ausdrücklich erinnert er die Biolog:innen daran, auf Fachvokabular („the alphabet“) zu verzichten: „Refrain from using the alphabet. Most of us in this room, including myself, are laypeople. We don’t understand your alphabet. So you will spell it out for us, so that we know exactly what you’re talking about, because we are here to listen. Thank you.“ (min 0:54–1:15).

Eine Stimme aus dem Off stellt die Expert:innen der Reihe nach vor und fasst deren Stellungnahmen zusammen. Mark Ptashne betont in seinem Statement, dass das Risiko, einen pathogenen Organismus zu schaffen (und darin sei sich die Mehrheit der Molekularbiolog:innen einig), rein hypothetisch sei („purely hypothetical“). Daniel Branton stimmt Ptashne zu, dass die meisten Biolog:innen ein enormes Potenzial in der rDNA-Forschung sehen, räumt allerdings ein, dass diese Tatsache niemanden davon freispreche, äußerste Vorsicht walten zu lassen. Die Dritte im Bunde ist Maxine Singer, die als Vertreterin der NIH die Guidelines erläutert. Nachdem sich Maxine Singer den Fragen Velluccis gestellt und die Sorgfalt der NIH in der Risikobewertung von rDNA-Experimenten hervorgehoben hat, äußert sie ihr Unbehagen darüber, wie sie in der Presse dargestellt wurde: „I must say that I have been surprised recently to find myself being put among those who are not concerned. I have been concerned and I continue to be concerned. I feel that these guidelines are a very responsible response to that concern.“ (min 5:21–5:37).

Der Film ist so zusammengeschnitten, dass die Statements der Wissenschaftler:innen und die Gegenfragen Velluccis im Mittelpunkt stehen, der während der Anhörung sein rhetorisches Talent unter Beweis stellt. Mit seiner Rhetorik und Gestik unterstreicht Vellucci sein Selbstverständnis als Richter von Cambridge, der im Interesse der einfachen Leute („lay people“) handelt. Er ist der Meinung, dass Labore für P3- und P4-Experimente in unbesiedelten Gegenden und nicht in Cambridge – einer der am dichtesten besiedelten Gemeinden der USA – gebaut werden sollten. Rhetorisch gekonnt bombardiert er Mark Ptashne und Maxine Singer mit kritischen, teils suggestiven Fragen:

„One. Did anyone of this group bother at any time to write to the mayor and city council to inform us you intended to carry out these experiments in the city of Cambridge, and you just said that you had public hearings. You plan to use E. coli in your experiments. Do I have E. coli inside my body right now? That’s a question. Don’t answer [now, C.L.], but you may, as you go along. Does everyone in this room have E. coli inside their bodies right now? Can you make an absolute, one hundred percent certain guarantee that there is no possible risk which might arise from this experimentation? Is there zero risk of danger? Answer that question, later, too, please. Would recombinant DNA experiments be safer if they were done in a maximum security lab, a P4 lab, in an isolated, nonpopulated area of the country? Question. Would this be safer than using a P3 lab in one of the most densely populated cities in the nation? Question. Is it true that in the history of science mistakes have been made, or known to happen? Question. Do scientists ever exercise poor judgment? Question. Do they ever have accidents? Question. [applause] Do you possess enough foresight and wisdom to decide which direction the future of mankind should take? Question.“ (min 6:10–7:42)

Eine längere Sequenz zeigt einen Schlagabtausch zwischen Mark Ptashne und den Stadträt:innen Saundra Graham und David Clem. Clem hält es für einen Widerspruch, dass Ptashne einerseits behauptet, er wolle mithilfe von rDNA mehr über die genetische Struktur höherer Organismen herausfinden, andererseits offenbar genug weiß, um Sicherheitsrisiken ausschließen zu können (min 15:42–16:10). Er appelliert an den „common sense“ der Wissenschaftler:innen, solche Versuche in weniger dicht besiedelten Gebieten durchzuführen. Ironischerweise war es David Clem selbst, der wenige Jahre später als Immobilienmakler den Kendall Square zum globalen Zentrum der Biotech-Industrie transformierte (Scheffler 2023).

Auf den Schlagabtausch zwischen Ptashne und Clem folgt der dramatische Höhepunkt der Dokumentation. Bürgermeister Vellucci schlägt eine Resolution vor, derzufolge sämtliche rDNA-Experimente in Cambridge für zwei Jahre verboten werden sollen. Mark Ptashne entgegnet vehement: Sollte diese Resolution verabschiedet werden, müsste ein Großteil der Experimente in Harvard und am MIT gestoppt werden, da sich die Resolution auch auf Experimente unterer Sicherheitsstufen bezieht. Vellucci reagiert mit einer persönlichen Anekdote aus seiner Kindheit: „When I was a little boy I used to fish in the Charles River and I woke up one morning and I found millions of fish dead in the Charles River and you tonight tell me that you dump chemicals into the sewage system of Cambridge.“ (min 22:03–22:17). Velluccis Reaktion zeugt erneut von seinem rhetorischen Geschick, da er mit seiner Anekdote zur Emotionalisierung und Skandalisierung der rDNA-Forschung beiträgt. Schließlich einigt man sich auf ein sechsmonatiges Moratorium, das ausschließlich für P3- und P4-Experimente gilt.

Zum Ende der Dokumentation folgen die Stellungnahmen der Opposition, unter ihnen die Biologin Ruth Hubbard und der Biochemiker sowie Science for the People-Aktivist Jonathan King. Hubbard führt in ihrer Kritik an, dass sogenannte „containment facilities“ keinerlei Garantie dafür seien, dass genetisch veränderte Mikroorganismen nicht aus dem Labor ausbrechen. Denn diejenigen, die mit E. coli arbeiten, würden diese unweigerlich hinaustragen – „on their clothes, in their hair, on their skin, in their throats, and it will be communicated by them to their people.“ (min 23:19–23:26) Jonathan King weist auf die politische Natur der rDNA-Kontroverse hin und kritisiert (ähnlich wie David Clem), dass ein tiefer Interessenkonflikt bestehe, wenn die NIH sowohl für die Finanzierung als auch Regulierung von rDNA-Experimenten zuständig seien: „Those guidelines are like having the tobacco industry write guidelines for tobacco safety.“ (min 29:09–29:14) Der Abspann fasst die weiteren Ereignisse der Cambridge-Kontroverse zusammen, die schlussendlich in der *Biosafety Ordinance* vom 7. Februar 1977 mündeten.

Aus den Oral-History-Interviews mit Ruth Hubbard und Mark Ptashne geht hervor, dass Charles Weiner seine Videoaufnahmen auch als Korrektiv zu den dürftigen Mitschriften verwendete, die während der Anhörung von Stenografen des City Council angefertigt wurden. Gegenüber Ruth Hubbard verriet er:

„Because one thing that we have is a poor transcript that the City Council who hired stenographers had made. And when we checked that against our videotape for the portions that we have tapes of, the transcript was filled with inaccuracies. So I thought it best if we get as many statements in the record as possible.“^31^

*Hypothetical Risk* ist ein ausgezeichnetes Lehrstück für die Wechselwirkung von Wissenschaft und Politik: Zum einen verdeutlicht der Film die Bedeutung von Rhetorik und politischer Inszenierung während der Cambridge-Kontroverse. Vellucci scheute nicht davor zurück, die potenziellen Gefahren des P3-Labors dramatisch auf die Spitze zu treiben und Mark Ptashne in die Ecke zu drängen. Zum anderen demonstriert die Dokumentation, dass innerhalb der Scientific Community keineswegs Konsens über die Gefahren der rDNA-Forschung herrschte. Ruth Hubbard und Jonathan King hielten die Risiken keinesfalls nur für hypothetisch; sie insistierten, dass es sich bei dieser Frage um eine genuin politische handle. Wie kaum ein anderes Zeugnis der Oral-History-Sammlung stellte der Film alte Vorstellungen von neutraler und wertfreier Wissenschaft auf den Kopf. Zugleich trugen die Aufnahmen zur Überhöhung der Cambridge-Kontroverse bei, die bis heute als dramatischer Höhepunkt der US-amerikanischen Gentechnikdebatte gilt.

Nicht nur die Oral-History-Interviews, auch die Dokumentation *Hypothetical Risk* fußten auf der Idee von Zeug:innenschaft: Studierende und Forschende der Technology Studies sollten sich – unabhängig von Autoritäten – ihr eigenes Urteil über die ethischen, sozialen und politischen Folgen der rDNA machen. Über das Medium Film wurden sie selbst zu Zeug:innen der Anhörung. Doch Anspruch und Wirklichkeit klafften auseinander, denn bei der Dokumentation handelte es sich um einen selektiven, kuratierten Zusammenschnitt eines historischen Ereignisses. Im Folgenden wird erörtert, inwiefern die Oral-History-Sammlung zur Schaffung neuer Mythen beitrug.

## Neue Mythen schaffen: Asilomar, Cambridge und Frankenstein

### Asilomar versus Cambridge

Die eingangs erwähnte Dokumentation *From Controversy to Cure* führt vor Augen, dass Cambridge heute einen festen Platz in der Geschichte der Gentechnik einnimmt – als Kulminationspunkt einer Auseinandersetzung zwischen Wissenschaft, Politik und Öffentlichkeit. James Watson und John Tooze hielten die Cambridge-Ereignisse in ihrem Quellenband *The DNA Story: A Documentary History of Gene Cloning* in dramatischen Bildern fest (Watson & Tooze 1981). Obwohl Charles Weiner den Anspruch hatte, mithilfe seiner Oral-History-Sammlung den gängigen Mythos einer objektiven, neutralen Wissenschaft zu widerlegen, trug seine Sammlung (zumindest indirekt) zur Mythenbildung um Cambridge bei. Während Asilomar in der Wissenschaftsgeschichte und den STS als Ort der wissenschaftlichen Selbstregulierung und strategischen Geheimhaltung gilt, ging Cambridge als Ort der öffentlichen Partizipation, Verantwortung und Transparenz in die Geschichte ein. Rae Goodell schreibt dazu: „The Cambridge experience was largely regarded as not only unprecedented but successful. The process was considered democratic and the outcome reasonable, a victory for public participation in science policy decisions.“ (Goodell 1979: 37). Ebenso charakterisierte der Wissenschaftshistoriker Robin Scheffler die öffentlichen Anhörungen in Cambridge jüngst als „unprecedented act of banning a scientific technique“ (Scheffler 2023). Sheldon Krimsky schreibt in *Genetic Alchemy*, dass der eigentliche Erfolg der Cambridge-Episode darin bestand, neue, demokratische Wege der Konsensfindung zu beschreiten:

„There was far more at stake than rDNA research. A decision-making apparatus was on trial. Should the citizenry defer decisions on the risks and benefits of new technologies to scientific experts? And if not, is there another way? What is most significant about the Cambridge episode is not the outcome, but ‚the other way‘.“ (Krimsky 1982: 298)

Doch bereits 1979 erkannte Rae Goodell, dass die Cambridge-Kontroverse beinahe einem Mythos gleichkam, der für widersprüchliche Errungenschaften stand: „Later, as the Cambridge legend grew, it took on mythical, and even contradictory dimensions: Cambridge pointed up the potential for effective public participation in science or Cambridge pointed up the potential for dangerous public meddling in science“ (Goodell 1979: 37). Während die einen in Cambridge die Vision einer aktiven Bürger:innenbeteiligung verwirklicht sahen, warnten andere vor einer gefährlichen Einmischung von Laien in forschungspolitische Entscheidungen. Da Weiners Oral-History-Sammlung die wesentliche Quellengrundlage für die wissenschaftshistorisch und STS-informierten Abhandlungen zur rDNA-Kontroverse in den USA bildete, trug sie zur Mythenbildung um Cambridge bei. Im Vorwort zu ihrem Opus Magnum *Molecular Politics* (1994) dankt Susan Wright explizit Charles Weiner in seiner Voraussicht, die rDNA-Debatte umfassend zu dokumentieren:

„Especially for the early period of policy formation I also want to acknowledge the importance of the materials drawn together by Charles Weiner and deposited in the MIT Institute Archives. In using this rich collection of documents, interviews, and tapes, I have benefited from his foresight in documenting the genetic engineering issue and related policy decisions as these evolved in the 1970s.“ (Wright 1994: xix)

Die Darstellung der Cambridge-Ereignisse in der Historiografie entsprach nur teilweise der Realität: Erstens war die Bildung von Bürgerkomitees keineswegs neu in Cambridge, sondern bereits vorher gängige Praxis. Doch im Gegensatz zu anderen Bürgerkomitees fehlte es dem Cambridge Experimentation Review Board (in dem neben einer Krankenschwester und einem Sozialarbeiter auch eine Nonne saß) an biologischer und technischer Expertise, um eine entsprechende Risikoeinschätzung vornehmen zu können (Goodell 1979: 39). Zweitens war der Einfluss des Stadtrats auf den Entscheidungsprozess weitaus geringer als in der Presse und Historiografie dargestellt. Die an der Harvard University intern geführte Debatte um das P3-Labor wurde auf den Stadtrat verlagert, wobei ähnliche Akteur:innen und Argumente die Debatte weiterhin dominierten. Wie Barbara Culliton zu Recht bemerkte, entsprach die öffentliche Diskussion um rDNA einer Debatte „between two scientific camps slugging it out in public“ (Culliton 1976: 274). Man muss bedenken, dass sich Harvard zum Zeitpunkt der Anhörungen schon kurz vor der Bewilligung der neuen Laborräume befand (Goodell 1979: 38). Um die öffentliche Meinung zu beeinflussen, setzten Harvard und MIT eine regelrechte PR-Kampagne in Gang, indem sie Koryphäen des Fachs zu den Anhörungen einluden und Nobelpreisträger wie James Watson und Paul Berg mobilisierten, sich per Brief an Vellucci zu wenden (Goodell 1979: 38). Besonders Watson hatte sich als geschickter forschungspolitischer Stratege bewiesen: Während der „War on Cancer“-Kampagne konnte er die Nixon-Regierung von der Bedeutung der Molekularbiologie als Fundament biomedizinischer Forschung überzeugen und erhebliche Geldsummen für die Krebsforschung am Cold Spring Harbor Laboratory akquirieren (Scheffler 2019: 148–166).

Drittens war die Opposition gegenüber dem geplanten P3-Labor keineswegs so geschlossen, wie es spätere Darstellungen suggerierten. Mitglieder von Science for the People standen nur lose mit Biolog:innen wie Ruth Hubbard und George Wald in Kontakt, die als unabhängige Expert:innen auftraten. Die oppositionellen Biolog:innen konnten nur wenig mit der teils polemischen Kritik der Politiker:innen, insbesondere Velluccis, anfangen. Wiederum andere Biolog:innen hielten sich mit ihrer Kritik zurück, da sie eine Stigmatisierung als linke „Spinner“ (*kooks*) oder Isolierung innerhalb ihrer Community befürchteten. „In short, the critics had no institutional base to provide support and no matrix for organization“, so das Fazit von Rae Goodell (dies. 1979: 38). Auf die Frankenstein-Rhetorik Velluccis zurückgreifend resümierte sie: „And so the legendary Cambridge chimera appears to have been a relatively familiar beast, a combination of traditional lay and scientific regulatory mechanisms.“ (Goodell 1979: 41). Viertens ergab die politische Rahmung der Cambridge-Kontroverse als ein Konflikt zwischen links-progressiven und rechts-konservativen Kräften nur wenig Sinn. In einem Interview mit Charles Weiner betonte George Wald:

„The paradox is in the universal feeling that it’s just the other way around, that the people who want to go ahead [with recombinant DNA research, C. L.] are thought of as on the right, and the people who are trying to stop them are universally thought of as on the left. The expressions of that situation politically are kind of interesting. For example, a relatively radical scientific group, such as Science for the People is bitterly opposed to the recombinant DNA business and makes no bones about it. [It is] one of their principal concerns. And from the other side, it’s strange that some people who are in the field and want to get on with it, resent deeply the implication that they are on the right since their self-image is so much on the left.“^32^

Wie wenig sinnvoll eine Unterteilung in „linke“ rDNA-Gegner:innen und „rechte“ rDNA-Befürworter:innen ist, zeigt sich an Mark Ptashne, dem Initiator des P3-Labors: Obwohl er von Kritiker:innen als Vertreter einer interessengeleiteten Wissenschaftselite gebrandmarkt wurde, war es Ptashne höchstpersönlich, der 1971 mit Science for the People nach Nordvietnam gereist war. Das von Richard Levins (1930–2016) – einem überzeugten Marxisten – geleitete Projekt wurde sendungsbewusst „Science for Vietnam“ genannt. Das Ziel war es, in der Demokratischen Republik Vietnam eine neue, an sozialistischen Ideen orientierte Forschung aufzubauen, basierend auf einem Curriculum, das sich mit den sozialen und politischen Implikationen von Wissenschaft auseinandersetzte. Ptashne referierte in Hanoi über Molekularbiologie und Genetik, andere über Ökologie und öffentliche Gesundheit. Am 15. Mai 1971 fand in Boston eine gleichnamige Konferenz statt, auf der Ptashne und Noam Chomsky über ihre Erfahrungen in Hanoi berichteten (Ptashne 1971: 19–23). Dass Wissenschaft und Politik untrennbar miteinander verwoben sind, daran schien auch Ptashne keinen Zweifel zu haben. In einem Interview mit Charles Weiner machte Ptashne keinen Hehl daraus, dass er und seine Kolleg:innen mit der Gordon Conference und dem Singer-Söll-Letter 1973^33^ ein politisches Statement setzen wollten: „I mean, this was the great event where scientists were showing how responsible they were and so on. And it’s more of a political statement than anything, in retrospect. In retrospect, one realizes how much of one’s so-called ‚rational‘ decisions are basically political or emotional.“^34^

In der Historiografie verfestigte sich ein Narrativ, das Asilomar und Cambridge gegeneinander ausspielte und jeweils einem Extrem zuordnete: Selbstregulierung versus Partizipation, Geheimhaltung versus Transparenz, Eigeninteressen versus Verantwortung, Hinterzimmer-Deals versus Öffentlichkeit. In der Molekularbiologie gilt Asilomar bis heute als Meilenstein, als „Woodstock“ der Biologie (vgl. Barinaga 2000): Eine Konferenz, die erfolgreich zwischen der Skylla der realen Gefahr und der Charybdis der Überregulierung vermitteln konnte (Hurlbut 2015: 126). Nicht nur in der Molekularbiologie, sondern in den Lebenswissenschaften allgemein transzendierte Asilomar zum Erinnerungsort, aus dem wissenschaftlich-technische Imaginationen für die Zukunft abgeleitet wurden (ebd.: 126–150). Biolog:innen erinnerten immer wieder an die legendären „Asilomar moments“, um für die Selbstregulierung der Wissenschaft einzutreten und Wissenschaft gegenüber Politik, Gesellschaft und Recht als progressive, dynamische und zukunftsweisende Kraft zu verklären (vgl. Schäfer & Low 2014).

In der Wissenschaftsgeschichte und den STS wurde die Asilomar-Konferenz dagegen überaus kritisch bewertet: Sie stand für den wohlkalkulierten Versuch, die rDNA auf ein rein technisches Problem zu reduzieren, ethische Fragen auszuklammern und Politik sowie Öffentlichkeit von der Regulierungsfrage auszuschließen. Asilomar schien die Geheimhaltungspolitik einer interessengeleiteten Wissenschaftselite zu verkörpern (vgl. Botelho 2019; de Chadarevian 2005; Jasanoff 2005; Gisler & Kurath 2011; Schäfer & Low 2014). Susan Wright konstatierte sogar, dass die Definition der rDNA als rein technisches Problem in Asilomar ihren Ausgang nahm und in der Folge zum „Dogma“ unter Molekularbiolog:innen wurde (Wright 1994: 159). Daran anknüpfend warf Charles Weiner Asilomar einen „technical fix“ vor – ein Vorwurf, den auch Sheldon Krimsky und Science for the People erhoben: „The recombinant DNA issue was defined as a technical problem to be solved by technical means, a technical fix“ (Weiner 2001: 211; Krimsky 1982: 15).

Gegenüber Asilomar erschien die Cambridge-Episode wie eine Kontrastfolie: In ihr schien die Vision einer demokratischen und partizipativen Wissenschaft verwirklicht. Doch weder Asilomar noch Cambridge waren eindeutig dem Extrem der Geheimhaltung oder Partizipation zuzuordnen – in beiden Fällen müssen Mythos und Wirklichkeit voneinander getrennt werden. Wie wir nun sehen, vermischten sich in der Cambridge-Kontroverse biowissenschaftliche mit fiktionalen Diskursen, die sich im Bild des Frankenstein-Monsters kristallisierten.

### Frankenstein at Harvard

Die Cambridge-Kontroverse steht bis heute in einem engen assoziativen Zusammenhang mit Frankenstein, dem legendären Wissenschaftler aus Mary Shelleys (1797–1851) gleichnamigen Roman, dessen aus Leichenteilen geschaffenes Monster zur Ikone der Popkultur wurde (Morowitz 1979; Isaacs 1987; Turney 1998; Ziolkowski 1981).^35^ Treffend bemerkt Jon Turney in seinem Buch *Frankenstein’s Footsteps: Science, Genetics and Popular Culture*: „So the *Frankenstein* myth was certainly invoked in some national commentaries. But the shadow of Frankenstein was perhaps most visible in one particular manifestation of the recombinant DNA debate, in Cambridge, Massachusetts“ (Turney 1998: 195). Dass die Cambridge-Kontroverse mit Frankensteins Monster in Verbindung gebracht wurde, ging auf Alfred Vellucci zurück, dessen Schlagabtausch mit den Harvard-Biolog:innen in der Dokumentation *Hypothetical Risk* verewigt wurde. In einem längeren Monolog mahnte Vellucci:

„I have made some references to Frankenstein over the past week and some people think, this is all a big joke, but that was my way of describing: What happens when genes are put together in a new way? This is a deadly serious matter, Sir, Madam, Harvard University. This is a serious matter. It is not a laughy matter, please believe me. […] If worse comes to worse we could have a major disaster on our hands. I guarantee everyone in this room that if that happens no one will be laughing then.“ (min 7:54–8:42)

In einem Interview mit Rae Goodell vom 9. Mai 1977 gestand Vellucci, dass er den Frankenstein-Vergleich aus strategischen Gründen gewählt hatte: „I thought Frankenstein would kind of shake them up [i.e. the press], and it would get news media to print. They were never satisfied – the print, TV, magazines – were never satisfied unless I gave them that touch of Frankenstein. So I played Frankenstein to the high heavens.“^36^ Offenbar ahnte Vellucci, dass der Frankenstein-Vergleich zur Skandalisierung der Cambridge-Ereignisse beitragen und die Aufmerksamkeit der nationalen Medien erregen würde (Vellucci 1977).

Velluccis Frankenstein-Szenario rekurrierte auf eine längere ideengeschichtliche Tradition der Kritik an wissenschaftlicher Hybris im Kontext biogenetischer Eingriffe (vgl. Turney 1998). Besonders in der Frühphase der rDNA-Debatte war Frankenstein ein beliebter Referenzpunk für Kritiker:innen, um latente Ängste vor der Erschaffung, unkontrollierbaren Vermehrung und Pathogenität transgener Organismen auszudrücken (Turney 1998: 192–197; Isaacs 1987: 80–104). Gentechnik-Kritiker:innen der ersten Stunde, unter ihnen Erwin Chargaff, Robert Sinsheimer und Jeremy Rifkin, sprachen bewusst von „genetic cloning“, „genetic meddling“ oder „genetic engineering“, um rDNA-Technologie mit düsteren Szenarien von Eugenik, Menschenzüchtung und genetischer Optimierung zu verknüpfen (Chargaff 1976; Sinsheimer 1975). Der Biochemiker und Schriftsteller Erwin Chargaff war einer der ersten, der den Frankenstein-Vergleich in einem öffentlichen Brief in *Science* verwendete, um vor möglichen Gefahren der rDNA zu warnen, insbesondere, wenn bei den Experimenten E. coli-Bakterien als Modellorganismen verwendet wurden: „If Dr. Frankenstein must go on producing his little biological monsters and I deny the urgency and even the compulsion why pick E. coli as the womb?“ (Chargaff 1976: 938).

**Abb. 3:**
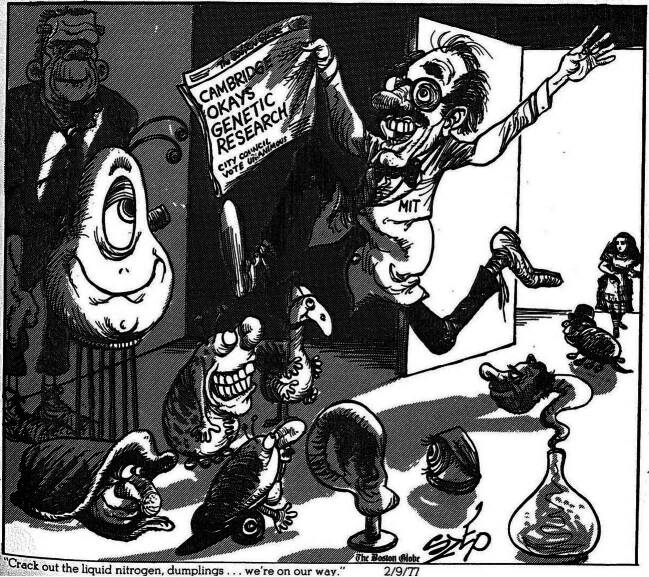
Karikatur aus dem *Boston Globe* (09.02.1977)

Dass Velluccis Frankenstein-Vergleich überaus wirkmächtig war, spiegelt sich in der zeitgenössischen Presseberichterstattung wider. In Anlehnung an Velluccis Worte titelte der *Washington Star* am 16. Juni 1976: „Is Harvard the proper place for Frankenstein tinkering?“ (Anonym 1976); *The Real Paper* aus Boston schrieb am selben Tag: „City to Probe Harvard Frankenstein-Lab“, während es im *Boston Herald* hieß: „Cambridge officials fear Harvard lab ‚monster‘“. Als das P3-Labor ein halbes Jahr später bewilligt wurde, setzte die Londoner *Times* die Schlagzeile „Frankenstein project given go-ahead in US“ auf ihre Titelseite.^37^ Eine Karikatur im *Boston Globe* vom 9. Februar 1977 zeigt den Molekularbiologen David Baltimore, der jubelnd und mit einer Tageszeitung in der Hand sein Labor betritt, auf der die Headline steht: „Cambridge okays genetic research“ (Abb. 3). Die Karikatur zeigt Baltimore umzingelt von im Labor geschaffenen Kreaturen, unter anderem auch dem Frankenstein-Monster: „Crack out the liquid nitrogen, dumplings … we’re on our way“, lautet die Bildunterschrift.^38^

Frankenstein-Bilder fanden auch Eingang in den wissenschaftlichen Diskurs der 1970er und 80er Jahre: Der Biologiehistoriker Everett Mendelsohn hielt 1979 am Zentrum für Interdisziplinäre Forschung in Bielefeld einen Vortrag über die Cambridge-Kontroverse mit dem schillernden Titel „Frankenstein at Harvard: The Public Politics of Recombinant DNA Research“ (Mendelsohn 1982, 1984). Mendelsohns Vortrag ging aus einer Studie zu den ethischen und sozialen Implikationen der rDNA-Forschung hervor, die er für die National Commission for the Protection of Human Subjects of Biomedical and Behavioral Research durchgeführt hatte. Mendelsohn erkannte in der Cambridge-Kontroverse und ihren Nachwehen zwei parallele Entwicklungen: Einerseits stießen traditionelle Kontroll- und Regulierungsmechanismen universitärer Forschung im Falle der rDNA an ihre Grenzen und wurden durch öffentliche Kontrollorgane ersetzt; andererseits fand die rekombinante Forschung rasch Anschluss an den industriellen Sektor, wo sie kommerziellen Regeln und Normen folgte (Mendelsohn 1984: 331). Mendelsohn brachte es auf den Punkt: Was Mitte der 1970er Jahre in Cambridge als Frankenstein-Hysterie begonnen hatte, mauserte sich binnen weniger Jahre zum regelrechten Biotech-Boom (vgl. Hughes 2001; Rasmussen 2014).

Die Vermischung von biowissenschaftlichen, fiktionalen und utopischen beziehungsweise dystopischen Diskursen war keineswegs ein Spezifikum der frühen Gentechnikdebatte: Molekularbiologie, Kybernetik und Raumfahrt regten in den späten 1960er Jahren utopische Diskurse über Klone, Cyborgs und Transhumanismus an, die an die Eugenik des Fin de Siècle anknüpften, dabei jedoch nicht auf die Abwehr von Degeneration und Dekadenz zielten, sondern die biotechnische Transformation und Überwindung des Menschen anstrebten (Brandt 2009: 258–261). In den 1970er Jahren erfassten jene Utopien auch die Debatte um rDNA, was sich unter anderem in der Rezeption des Science-Fiction-Thrillers *The Andromeda Strain* äußerte, der 1971 in die Kinos kam (Campos 2021: 152–155). Der Film, beruhend auf einer Romanvorlage von Michael Crichton, handelt von einer Raumsonde des US-amerikanischen Militärs, die in New Mexico einschlägt und eine Epidemie auslöst. Als Verursacher der Epidemie wird wenig später „Andromeda“ identifiziert – infektiöse Mikroben aus dem Weltall, die organische und anorganische Materie zersetzen. Es stellt sich bald heraus, dass das Militär gezielt nach extraterrestrischen Organismen gesucht hatte, um aus ihnen biologische Waffen herzustellen. In der öffentlichen, teils reißerischen Berichterstattung über Asilomar blieb die Sorge vor einer tödlichen Andromeda-Invasion ein konstanter Referenzpunkt; Andromeda war das mikrobiologische Pendant zum Frankenstein-Monster (Campos 2021: 159–163; Cobb 2022: 90).

Auch die Cambridge-Ereignisse wurden von Andromeda-Ängsten überschattet. Die Stadträtin Barbara Ackermann gestand in einem Interview mit Rae Goodell, dass ihre Skepsis gegenüber den Laborplänen in Harvard durch *Andromeda Strain* geschürt wurde, den sie kurz zuvor im Fernsehen gesehen hatte (Ackerman 1977).^39^ Der *Boston Star* schrieb am 16. Juni 1976: „It seems science fiction has arrived unannounced to Cambridge – the vision of the future has been dumped on our city.“ (Boston Star, 16.06.1976). Analog zur Debatte um das Klonen vermischten sich in der Kontroverse um rDNA wissenschaftliche mit fiktionalen und dystopischen Diskursen (vgl. Brandt 2009). Dass diese Science-Fiction-Szenarien zumindest für kurze Zeit reale Ängste auslösten, illustriert ein Brief Velluccis an Philip Handler (Präsident der NAS) vom Mai 1977, in dem Vellucci mit Sorge von einer „strange, orange-eyed creature“ berichtet, die angeblich in Neuengland gesichtet worden sei.^40^

Vellucci war es gelungen den Frankenstein-Vergleich medienwirksam zu platzieren und den Cambridge-Ereignissen so mehr Sichtbarkeit zu verleihen. Die Vermischung von biowissenschaftlichen und fiktionalen Diskursen, die besonders die frühe Phase der rDNA-Debatte prägten, kristallisierte sich in der Dystopie eines von Menschenhand geschaffenen Monsters. Durch das Zusammenspiel von Presseberichterstattung, Oral History und filmischer Dokumentation fand das Frankenstein-Bild allmählich Eingang in ein historisches Narrativ, das bis heute fortwirkt. Nicht ohne Grund überschrieb Joe McMaster in seiner eingangs erwähnten Dokumentation die Cambridge-Kontroverse mit dem Titel „Frankenbugs?“.

## Fazit: Mythen und Gegenmythen

Nirgends halten sich Mythen so hartnäckig wie in der Wissenschaft: Der junge Newton soll das Gravitationsgesetz angeblich durch einen herunterfallenden Apfel im elterlichen Garten entdeckt haben und Archimedes den Brennspiegel, als er römische Schiffe bei einer Belagerung mit einem Spiegel in Brand setzte. Archimedes war es auch, der angeblich nackt durch Syrakus lief und „Heureka!“ rief, nachdem er in der Badewanne sitzend das Gesetz der Auftriebskraft entdeckt hatte (heute als Archimedisches Prinzip bekannt). So unterschiedlich diese Mythen auch sind, sie haben eines gemeinsam: Sie legitimieren eine wissenschaftliche Disziplin, indem sie legendäre Ahnen, Ursprungsszenen und Gründungsorte schaffen.

In der Geschichte der Gentechnik war dies nicht anders. Die Dokumentation *From Controversy to Cure* verdeutlicht, dass die Cambridge-Kontroverse von 1976 zum Mythos der US-amerikanischen Gentechnikdebatte geworden ist – zum dramatischen Höhepunkt einer Auseinandersetzung zwischen Wissenschaft, Politik und Öffentlichkeit, aus der letztlich ein Welterfolg hervorging. Es ist der Recombinant DNA History Collection von Charles Weiner zu verdanken, dass die Cambridge-Ereignisse bis heute so gut dokumentiert sind und einen festen Platz in der Historiografie und Erinnerungskultur der Lebenswissenschaften einnehmen. Neben Thomas Kuhns Sources for History of Quantum Physics war Weiners Oral-History-Sammlung eine der ersten und größten ihrer Art in den USA.

Jenseits ihres dokumentarischen Charakters hatte die Sammlung auch eine politische und aufklärerische Mission: Zum einen sollte sie den Mythos einer neutralen, objektiven und wertfreien Wissenschaft widerlegen; zum anderen schloss sie an den demokratischen Anspruch der Oral History an, indem sie nicht nur bekannten Molekularbiolog:innen eine Stimme gab, sondern auch Vertreter:innen aus Politik, Wirtschaft, Journalismus, Umweltverbänden und Oppositionsgruppen. Zu den Interviewpartner:innen zählten der Molekularbiologe Paul Berg ebenso wie die Lokalpolitikerin Barbara Ackerman, die Wissenschaftshistorikerin Susan Wright und der Wissenschaftsphilosoph und Science-Policy-Experte Sheldon Krimsky. Die Recombinant DNA History Collection folgte dabei dem subversiven Ziel, hegemoniale Erzählungen von Asilomar durch Gegennarrative herauszufordern. Ihr Herzstück war die Dokumentation *Hypothetical Risk*, die Studierenden vor Augen führen sollte, dass Wissenschaft keine neutrale Erkenntnissuche sei, sondern ein politisches und interessengeleitetes Phänomen. Trotz dieser Ziele hatte die Sammlung einen Nebeneffekt: Sie verstärkte bestehende Biases und trug aktiv zur Schaffung neuer Mythen bei. Cambridge ging als Vorbild für öffentliche Partizipation in forschungspolitischen Entscheidungen und damit als Gegenentwurf zu Asilomar in die Annalen der Wissenschaftsgeschichte ein, obwohl diese Zuschreibung mehr Mythos denn Wirklichkeit entsprach – dies erkannten auch schon Zeitgenoss:innen (Goodell 1979).

Die Recombinant DNA History Collection war Ausdruck einer wissenschaftshistorischen Trendwende der 1970er Jahre, die Wissenschaft als soziales, politisches und von Machtstrukturen durchzogenes Phänomen erforschte. Nicht nur herrschaftskritische Organisationen aus dem Umkreis der Neuen Linken wie Science for the People vertraten diesen neuen Wissenschaftsbegriff, sondern auch ein Feld, das später als Science and Technology Studies (STS) bekannt werden sollte. Für Dorothy Nelkin, eine Pionierin der STS in den USA, entwickelte sich die rDNA zur „atomic bomb“ der 1970er Jahre: „a symbol of concerns about science and its consequences.“ (Nelkin 1979: 180) In der Cambridge-Kontroverse kristallisierten sich ihr zufolge Wertekonflikte um Wissenschaft zwischen Experten, Laien und Öffentlichkeit (Nelkin 1978a, b). Wie soll Wissenschaft reguliert werden? Wer soll sie regulieren, und wer hat die Autorität, über Regulierungsfragen zu entscheiden? Dorothy Nelkin, Sheldon Krimsky, Charles Weiner, Susan Wright und andere erhoben Cambridge zum regelrechten *role model* für öffentliche Partizipation in Science-Policy-Prozessen. Damit trugen sie, wie Rae Goodell bereits 1979 erkannte, zu einer fast mythologischen Überhöhung der Cambridge-Episode bei (Goodell 1979: 37). Hinzu kamen Politiker wie Alfred Vellucci, die die Cambridge-Ereignisse medienwirksam platzierten und für Wahlkampfzwecke instrumentalisierten.

Die Cambridge-Kontroverse hatte für eine neue Generation von Wissenschaftler:innen, die von den machtkritischen Diskursen der Neuen Linken geprägt wurde, auch eine sinn- und identitätsstiftende Funktion: Im Gegensatz zu Asilomar verkörperte Cambridge die Vision einer verantwortungsvollen, demokratischen und partizipativen Wissenschaft, die sich den Machtstrukturen des sogenannten *Scientific Establishment* widersetzte.

## Dank

Dieser Aufsatz ist das Ergebnis lebhafter Diskussionen am Lehrstuhl für Wissenschaftsgeschichte der LMU München. Ich bedanke mich bei den Kolleginnen und Kollegen der DFG-Forschungsgruppe „Kooperation und Konkurrenz in den Wissenschaften“ (LMU) für wertvolle Kommentare zu einer früheren Fassung des Textes. Ein besonderer Dank gilt: Daniela Hettstedt, David Irion, Moritz Schlenker, Johannes Schuckert und Cora Stuhrmann sowie den anonymen Gutachter:innen. Weiterhin danke ich den Archivar:innen des MIT für die großzügige Bereitstellung der Oral-History-Interviews und weiterer Dokumente aus der Recombinant DNA History Collection. Zu guter Letzt durfte ich das Thema auf verschiedenen Konferenzen vorstellen, von denen ich sehr profitiert habe. Vielen Dank auch dafür.

## Archivbestände

- Charles Weiner Papers, Institute Archives and Special Collections, MIT Libraries.
- James Watson Papers, Cold Spring Harbor Laboratory Archives.
- Joshua Lederberg Papers, National Library of Medicine, NIH.
- Maxine Singer Papers, Library of Congress, Washington, D.C.
- Recombinant DNA History Collection, Institute Archives and Special Collections, MIT Libraries (o. J.).
- STS Program Files, Institute Archives and Special Collections, MIT Libraries.
- Sydney Brenner Papers, Wellcome Collection, London.

## Footnotes

1 McMaster, Joe (2019). From Controversy to Cure: Inside the Cambridge Biotech Boom. URL: https://www.controversytocure.mit.edu/.

2 Joe McMaster ist Senior Producer bei den MIT Video Productions, einem hauseigenen Produktionsteam, das Dokumentationen, Filme und Videos über das MIT und dessen Geschichte produziert. URL: https://mvp.mit.edu/.

3 Die NIH stufte rDNA-Experimente auf einer Risikoskala von P1 bis P4 ein (P3 entsprach demnach der zweithöchsten Risikostufe). Die Stufen sahen eine Mischung aus „physical“ und „biological containment“ vor. Die erste Kategorie stand für physische Sicherheitsmaßnahmen im Labor (Schutzkleidung, Luftabzug etc.), während sich die zweite auf die Organismen bezog, deren DNA bei den Experimenten transferiert wurde. Die Modellorganismen wurden so abgeschwächt, dass sie außerhalb des Labors nicht überleben konnten.

4 McMaster, Joe (2019). From Controversy to Cure. URL: https://www.controversytocure.mit.edu/.

5 Das Columbia Center for Oral History Research (ursprünglich: Columbia University Oral History Research Office) beheimatet das weltweit größte Oral-History-Archiv. Heute umfasst das Archiv circa 8.000 Interviews auf Tape und mehr als eine Million Transkriptseiten. Vgl. https://www.ccohr.incite.columbia.edu/.

6 Exemplarisch seien das Oral-History-Projekt Sources for History of Astrophysics (1976–80) sowie das International Project on the History of Solid State Physics (1979–92) genannt, in denen die gesamte Physik-Community, inklusive Laborpersonal, zu Wort kam (Doel 2003: 357).

7 Zu den wenigen Frauen, die an der Asilomar-II-Konferenz teilnahmen, gehörten Elena Nightingale (Medizin), Jane Setlow (Genetik), Maxine F. Singer (Biochemie), Artemis Simopoulos (Endokrinologie), Anna Marie Skalka (Virologie/Molekularbiologie). Zur Teilnehmerliste der Asilomar II-Konferenz vgl. Report of the Organizing Committee of the Asilomar Conference on Recombinant DNA Molecules, Sydney Brenner Papers, Wellcome Collection. URL: https://wellcomecollection.org/works/zfkcg9g3.

8 Interview von Craig Waddell mit George Wald, Cambridge/Massachusetts, 17.03.1988, George Wald Papers, Harvard University Archives, Box 112: 8.

9 Vgl. Brief von Ruth Hubbard an Daniel Branton, Cambridge/Massachusetts, 08.04.1976, Institute Archives and Special Collections, MIT Libraries. Zit. n. Krimsky (1982: 299): „Much more worrisome to my mind than the work with viruses is the work with recombinant DNA. Viruses are more or less known quantities and the Biohazards Committee can restrict work to those it deems reasonably safe – say as safe as walking into a crowded lecture hall during flu season. But recombinant DNA is another story, because nobody can predict how genes will act in the vicinity of genes they have not been next before […]. I am frankly terrified at the idea of playing evolution, because it is an exceedingly dangerous game whose rules we will not know until it is too late.“

10 Vellucci soll 1974 sogar den Vorschlag eingebracht haben, das Herzstück der Universität, den Harvard Yard, in einen öffentlichen Parkplatz umzuwandeln – ein Vorhaben, mit dem er sich seinen Harvard-kritischen Ruf sicherte (vgl. Winling 2018: 166).

11 James Watson an Alfred Vellucci, Cold Spring Harbor, 29.06.1976, CSHL Archives Repository, JDW/2/2/795/83. URL: https://libgallery.cshl.edu/items/show/39466; Paul Berg an Alfred Vellucci, Stanford University, 02.07.1976, Paul Berg Papers, Stanford University Libraries, Box 15. URL: https://profiles.nlm.nih.gov/101584580X113

12 Zuständig für die Auswahl der Bürger:innenvertreter war der City Manager von Cambridge James L. Sullivan. In einem Brief an Bürgermeister Vellucci betonte er, er habe nach neutralen Bürger:innen gesucht, die sich dem Thema in einer „unbiased manner“ näherten und für die die öffentliche Sicherheit an oberster Stelle stand. James L. Sullivan an Alfred Vellucci, Cambridge/Massachusetts, 06.08.1976, Maxine Singer Papers.

13 Ronald Doel verzeichnet für den Zeitraum von 1950 bis 1969 folgende wissenschaftshistorische Oral-History-Projekte in den USA und Kanada: Columbia University Oral History Research Office Collection (1948 bis heute); Sources for History of Quantum Physics, UC Berkeley (1961–73); Stennis Space Center History Project, NASA (frühe 1960er, fortgeführt in den frühen 1990ern); IEEE History Project (1968 bis heute). Für den Zeitraum von 1970 bis 1979: Smithsonian Oral History Collection (1973 bis heute); Recombinant DNA History Collection, MIT (1975–79); Institutional History of the National Research Council of Canada (1975–81); Sources for History of Modern Astrophysics, American Institute of Physics (1976–80); History of Modern Oceanography (1976–77); Women in Science and Engineering Collection, MIT (1976–77); International Project in History of Solid State Physics, MIT (1979–92). Vgl. Doel (2003).

14 Vgl. Nachruf des MIT auf Charles Weiner: https://news.mit.edu/2012/obit-weiner.

15 Draft of Technology Studies Program Plan, STS Program Files, Institute Archives and Special Collections, MIT Libraries, Box 1: 5: „The Technology Studies Program’s efforts in undergraduate education begin with an idea that has been basic to M.I.T. education for a long time: that undergraduates should be informed about the sources and implications of their future work. Our goal is to help students achieve a critical understanding of how scientific and technological activity is an integral part of culture; how the values and institutions of society affect that activity; how technological innovation bears on social choice; by what complex and personal processes change takes place; how such changes affect social structures, political and economic processes, individual consciousness, and aesthetic norms.“

16 Draft Technology Studies Program Plan, STS Program Files, Institute Archives and Special Collections, MIT Libraries, Box 1: 11.

17 Aufgelistet wurden: 1 Sony TC-110A AC/DC portable cassette recorder, 1 Sony BM-25 Transcriber with foot pedal backspacing, 1 Sony BE‑7 Tape Cassette Bulk Eraser, 1 Sony Rechargeable Battery Pack, 1 BM-25 Transcriber. William Speer an Nathan Sivin, Cambridge/Massachusetts, 17.04.1974, STS Program Files, Institute Archives and Special Collections, MIT Libraries, Box 1.

18 Daniel Kevles an Nathan Sivin, Caltech, 14.03.1974, STS Program Files, Institute Archives and Special Collections, MIT Libraries, Box 1.

19 Nathan Sivin hatte sich bei der Planung des Technology Studies Program mit Roy McLeod in Verbindung gesetzt, der an der Universität Sussex ähnliche Ziele verfolgte. Er begründete dort das Program in History and Social Studies of Science (HSSS) und gab mit David Edge (1932–2003) die Zeitschrift *Social Studies of Science* heraus – das erste und führende STS-Journal in Europa. Vgl. Nathan Sivin an Roy McLeod, 15.05.1974, STS Program Files, Institute Archives and Special Collections, MIT Libraries, Box 1.

20 Neben Charles Weiner und Rae Goodell führten Lynette Maloney und Mary Terrall einzelne Interviews durch. Für die Transkription der Interviews waren die Sekretär:innen Janet Billane, Ruth Davidson, Beth Gould und R. Archibald-Woodward zuständig. Vgl. Oral History Interviews, Recombinant DNA History Collection, Institute Archives and Special Collections, MIT Libraries.

21 Rae Goodell an Joshua Lederberg, Cambridge/Massachusetts, 16.07.1976, Joshua Lederberg Papers, National Library of Medicine, NIH. URL: http://resource.nlm.nih.gov/101584906X8907

22 Mit *Jason scientists* ist jene Gruppe von Physiker:innen gemeint, die nach dem Sputnik-Schock 1957 die US-amerikanische Regierung in kriegsstrategischen und Verteidigungsfragen beriet.

23 Der in Harvard lehrende Edward O. Wilson versuchte mit seiner Soziobiologie eine neue Disziplin zu begründen, die menschliches Sozialverhalten und gesellschaftliche Organisation auf evolutionsgenetische Faktoren zurückführte. Seine Gedanken dazu hielt er in seinem Opus Magnum *Sociobiology: The New Synthesis* (1975) fest.

24 Interview von Charles Weiner mit John Tooze, Cambridge/Massachusetts, 26.03.1976, Recombinant DNA History Collection, Institute Archives and Special Collections, MIT Libraries: 85.

25 Interview von Charles Weiner mit Ruth Hubbard, Cambridge/Massachusetts, 24.01.1978, Recombinant DNA History Collection, Archives and Special Collections, MIT Libraries: 51.

26 Charles Weiner et al. (1976). *Hypothetical Risk: Cambridge City Council’s Hearings on DNA Experimentation in Cambridge*. URL: https://cdn.libraries.mit.edu/dissemination/diponline/MC0100/39080024063874_1of1.mp4

27 Verantwortlich für den Schnitt waren Charles Weiner, Lynette Maloney und Mitchell Trachtenberg, die unter anderem auch die Zeitzeug:inneninterviews führten.

28 Cambridge City Council, Public hearing, order of events, press release, 03.06.1976, Institute Archives and Special Collections, MIT Libraries. URL: https://hdl.handle.net/1721.3/200721

29 Ebd.

30 Der Biochemiker Jonathan King schrieb 1977 im *New Scientist*: „The universities make very little contribution to the people of the city, either to the school system, physical surroundings, or local economy. In fact one of the primary roles of the universities has been to try to move blue collar people out of the city in order to build luxury housing to attract faculty.“ (King 1977: 635).

31 Interview von Charles Weiner mit Ruth Hubbard: 50.

32 Interview von Charles Weiner mit George Wald, 16./30.01.1978, Cambridge/Massachusetts, George Wald Papers, Harvard University Archive, Box 106: 31f.

33 Der Singer-Söll-Letter (1973) war an den Präsidenten der National Academy of Science gerichtet und wies auf mögliche Gefahren transgener Organismen hin. Ferner plädierte er für ein Expert:innenkomitee, das Richtlinien für entsprechende Experimente erarbeiten sollte.

34 Interview von Charles Weiner mit Mark Ptashne, Cambridge/Massachusetts, 01.02.1978, Recombinant DNA History Collection, Institute Archives and Special Collections, MIT Libraries: 5.

35 Vgl. Harvard’s „Frankenstein“: The 70s Controversy over Mixing DNA. URL: https://www.huffpost.com/entry/harvards-frankenstein_n_6979452.

36 Interview von Rae Goodell mit Alfred Vellucci, Cambridge/Massachusetts, 09.05.1977, Recombinant DNA Oral History Collection, Archives and Special Collections, MIT Libraries: 39.

37 Vgl. *The Washington Star* (16.06.1976); *The Real Paper* (16.06.1976); *Boston Herald* (16.06.1976); *Times* (09.02.1977).

38 *Boston Globe* (09.02.1977).

39 Interview von Rae Goodell mit Barbara Ackermann, Cambridge/Massachusetts, 16.03.1977, Recombinant DNA History Collection, Archives and Special Collections, MIT Libraries: 6.

40 Alfred Vellucci an Philip Handler, Cambridge/Massachusetts, 16.05.1977. Abgedruckt in: Watson Tooze (1981: 206).
